# HIV Epidemic Appraisals for Assisting in the Design of Effective Prevention Programmes: Shifting the Paradigm Back to Basics

**DOI:** 10.1371/journal.pone.0032324

**Published:** 2012-03-01

**Authors:** Sharmistha Mishra, Sema K. Sgaier, Laura H. Thompson, Stephen Moses, B. M. Ramesh, Michel Alary, David Wilson, James F. Blanchard

**Affiliations:** 1 Department of Infectious Diseases Epidemiology, Imperial College, London, United Kingdom; 2 St. Michael's Hospital, University of Toronto, Toronto, Canada; 3 Global Health Program, Bill and Melinda Gates Foundation, New Delhi, India; 4 Department of Community Health Sciences, Centre for Global Public Health, University of Manitoba, Winnipeg, Canada; 5 Karnataka Health Promotion Trust, Bangalore, India; 6 URESP, Centre de recherche FRSQ du CHA universitaire de Québec, Université Laval, Québec, Canada; 7 Global HIV/AIDS Program, World Bank, Washington, D.C., United States of America; Vanderbilt University, United States of America

## Abstract

**Background:**

To design HIV prevention programmes, it is critical to understand the temporal and geographic aspects of the local epidemic and to address the key behaviours that drive HIV transmission. Two methods have been developed to appraise HIV epidemics and guide prevention strategies. The numerical proxy method classifies epidemics based on current HIV prevalence thresholds. The Modes of Transmission (MOT) model estimates the distribution of incidence over one year among risk-groups. Both methods focus on the current state of an epidemic and provide short-term metrics which may not capture the epidemiologic drivers. Through a detailed analysis of country and sub-national data, we explore the limitations of the two traditional methods and propose an alternative approach.

**Methods and Findings:**

We compared outputs of the traditional methods in five countries for which results were published, and applied the numeric and MOT model to India and six districts within India. We discovered three limitations of the current methods for epidemic appraisal: (1) their results failed to identify the key behaviours that drive the epidemic; (2) they were difficult to apply to local epidemics with heterogeneity across district-level administrative units; and (3) the MOT model was highly sensitive to input parameters, many of which required extraction from non-regional sources. We developed an alternative decision-tree framework for HIV epidemic appraisals, based on a qualitative understanding of epidemiologic drivers, and demonstrated its applicability in India. The alternative framework offered a logical algorithm to characterize epidemics; it required minimal but key data.

**Conclusions:**

Traditional appraisals that utilize the distribution of prevalent and incident HIV infections in the short-term could misguide prevention priorities and potentially impede efforts to halt the trajectory of the HIV epidemic. An approach that characterizes local transmission dynamics provides a potentially more effective tool with which policy makers can design intervention programmes.

## Introduction

As policy makers and programme planners consider how best to allocate limited resources to maximize the impact of their investments in HIV prevention [1], they require epidemic appraisals that provide accurate and timely guidance on the status and key epidemiologic drivers of ongoing local transmission [2], [3]. Epidemics often exhibit substantial geographical heterogeneity within countries [4], [5], [6], and the development of HIV prevention strategies is complicated by the need to balance the development of national prevention policy with the exigencies of decentralized programmes that aim to address epidemics at the local level [7]. However, appraisals and policy are often restricted to the national level and translated into an overarching prevention strategy that is applied uniformly across localities [3], [8]. The programmes then fall short of addressing local epidemiological situations, and are likely to generate an ineffective and inefficient response [7].

Epidemic appraisals must also correspond to programme objectives. Whereas treatment and support services require information on current distribution of HIV prevalence and incidence, prevention policy must be driven by considerations of how best to reduce HIV incidence in the long-term. The propagative nature of HIV epidemics and the heterogeneity in HIV acquisition and transmission between individuals and across subsections of a population result in a “dynamic topology” of these epidemics with respect to the epidemic trajectory, amplitude and distribution of prevalent and incident infections [9], [10]. Therefore, an effective prevention strategy that seeks to halt and reverse the course of an epidemic must be guided by information about the underlying epidemiologic drivers and proximal sources of new infections [9], [11].

Since the onset of the global HIV epidemic two main approaches for epidemic appraisal have been developed to guide HIV prevention strategies: 1) the “numerical proxy” method [12] and, 2) the “Modes of Transmission” (MOT) approach [13], [14], [15], [16]. The numerical proxy method categorizes epidemics on the basis of cross-sectional HIV prevalence thresholds using surveillance data [12]. In practice, prevalence estimates are usually derived from periodic surveys among women attending sentinel antenatal clinics (ANC) as a proxy for the general population [12], and among defined key populations at higher risk such as female sex workers (FSWs) and injecting drug users (IDUs). Epidemics are classified as ‘low level’ if HIV prevalence has not consistently exceeded 5% in any sub-population [12]. ‘Concentrated’ epidemics are those where the HIV prevalence is consistently greater than 5% in any key population but less than 1% in pregnant women [12]. ‘Generalized’ epidemics require that HIV prevalence persistently exceeds 1% in pregnant women [12]. Accordingly, HIV prevention would be prioritized to the key populations in a ‘concentrated’ epidemic and to the wider population in a ‘generalized’ epidemic [12].

The MOT approach involves predicting the number of new HIV infections that will occur in mutually exclusive risk-groups over a one-year period [13], [14], [15], [16]. The results of MOT analyses are now being used in a growing number of jurisdictions as a roadmap for HIV prevention, since they are intended to provide information on the current sources of new infections within the population and therefore, guidance on where prevention efforts should be prioritized [17], [18], [19]. First applied in 2002 [16], the MOT approach uses a static mathematical model that is simple to use but requires more data than the numerical proxy approach. It requires: (a) estimates of the size of behaviourally defined subgroups and their direct sexual partners; (b) the frequency of sexual acts per year in different types of partnerships; and (c) the current prevalence of HIV and sexually transmitted infections (STIs) in each subgroup. The theoretical basis for the MOT is the assumption that an uninfected individual's risk of acquiring HIV is a binomial function of their number of partners and contacts (or sex acts per partnership) and is dependent on the HIV prevalence among partners [13], [15].

Hence, the two traditional methods of HIV epidemic appraisal focus on either the current distribution of HIV prevalence or the current distribution of HIV incidence across subsections of a population. Yet despite their extensive use in guiding the design of prevention policies, the utility and limitations of these methods have never been empirically examined and questions remain about their validity in directing HIV prevention programmes [20]. Although relatively simple to apply, the prevalence thresholds employed by the numerical proxy method have never been validated and may not adequately distinguish between disparate epidemics types, nor provide sufficient detail about the underlying transmission dynamics to enable specific guidance about prevention priorities. Conversely, the MOT approach relies on data that are often not available [21], [22] or accurate [23]. Since both methods provide a ‘snapshot’ of an epidemic, they may discount the long-term trajectory of an epidemic and fail to identify epidemiologic drivers.

In this study, we empirically examine the utility and limitations of the numerical proxy and MOT model in diverse settings to determine how they compare with respect to HIV prevention policy implications at the national and sub-national levels. We focus our sub-national analyses on districts in India as a case study, since the country exhibits considerable heterogeneity in regional epidemics, and the extensive sub-national data required for this study has been collected at the district level. We demonstrate the limitations of the existing methods and recommend a paradigm shift in how we appraise HIV epidemics for the design of prevention programmes. We advocate for the development of approaches that are based on the population sexual structure and underlying transmission dynamics, such that epidemics are classified according to behaviours that enable HIV to persist in a geographic area in the long-term. We illustrate how such an approach might be applied at the local level, and how the results and HIV prevention policy implications compare to those provided by the numerical proxy and MOT approaches.

## Methods

### Numerical proxy and MOT appraisals

For an empirical analysis of findings from the numerical proxy and MOT approaches we first compared six countries with diverse epidemic characteristics: Uganda, Kenya, Nigeria, Peru, Thailand and India. MOT results for all countries except India were taken from published reports [13], [21], [22], [24], [25]. For India, we conducted the MOT analysis using established analytical approaches (The Workbook Method) as detailed previously [13], [15]. The population sexual structure follows the generic model provided in the Workbook Method [15]. Briefly, the population is divided into the following risk-groups: low-risk heterosexual (men and women reporting only one sexual partner in the year preceding the survey from which size estimates were obtained), FSWs, clients of FSWs, high-risk men who have sex with men (HR-MSM), injecting drug users (IDU), multiple partnerships (men and women reporting more than one sexual partner in the year preceding a given survey), and main partners or spouses of clients, HR-MSM, IDU, and individuals in multiple partnerships.

For sub-national comparisons, we examined six districts in India (Bagalkot, Belgaum, Ganjam, Shimoga, Sikar, and Varanasi), selected to reflect diversity within the country. To conduct the epidemic appraisals in India, we used the most recent input parameters available, obtained from general population surveys, key population biological and behavioural surveys, and key population mapping data (Text S1). Parameters for which district-level values were not available were assigned estimates from state- or national-level data.

To assess the sensitivity of the MOT to the quality of input parameters, we examined Belgaum district, using four data sources: (1) state-level estimates (i.e. we assumed district-level data was not available); (2) district-level estimates using general population and key population face-to-face interviews of self-reported behaviour; (3) district-level estimates using polling booth surveys [23] for population size estimates of clients and persons reporting multiple partnerships; (4) district-level estimates based on polling booth surveys [23] for most parameters, but with size estimates of the population of male clients of FSWs being derived indirectly, based on estimates of the size of the FSW population and the average number of clients per year (derived from the average number of clients reported by FSWs annually and frequency of FSW partnerships reported by clients). Details on data sources and parameter values, including estimates for the sensitivity analysis, are provided in Text S1 and Table S1.

### Transmission dynamics epidemic classification

**Figure 1:**
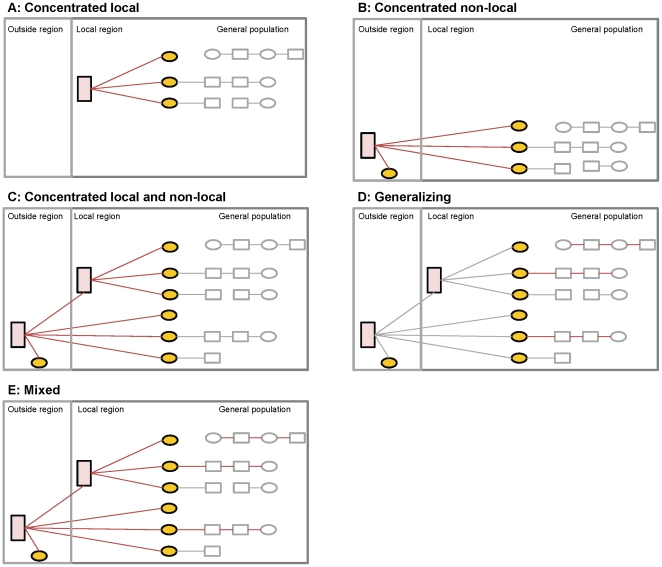
Epidemic typologies based on an alternative framework. High-risk groups (pink box) comprise of female sex workers, high-risk men who have sex with men, and injection drug users. Yellow circles indicate direct sexual partners of members of a high-risk group (for example, male clients). Grey boxes comprise the remainder of the general population. Red lines delineate sexual partnerships that contribute to emergence and persistence of HIV in the local community (epidemiologic drivers), such that in the absence of these partnerships, the epidemic would fail to establish.

**Figure 2:**
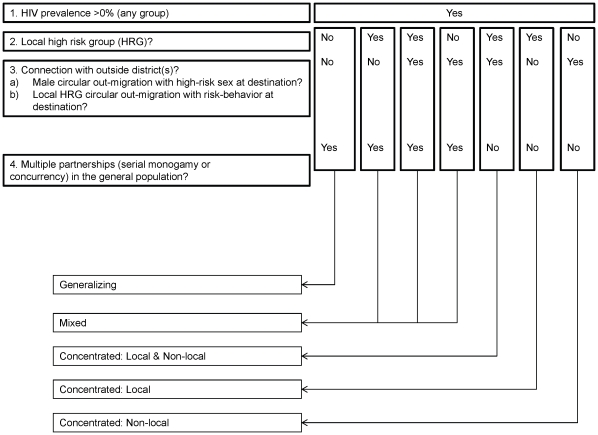
A proposed approach for assigning HIV epidemic typologies for the design of HIV prevention programmes. The epidemic drivers of concentrated epidemics are networks of HRGs, whereas multiple partnerships enables HIV to be sustained in the generalizing epidemic. In the mixed epidemic, there is substantial contribution from both the HRGs and the general population in sustaining HIV transmission. Int refers to intermediate.

To address long-term prevention goals, we developed an alternate approach that seeks to differentiate epidemics based on their underlying transmission dynamics and epidemic potential. This approach, which we have labelled “transmission dynamics epidemic classification” (TDEC) uses information about the population sexual structure and a decision-tree logic to broadly classify epidemics based on the likely contribution of identifiable higher-risk key populations to HIV emergence and persistence. The classification builds on early work by Wasserheit and Aral [9] that distinguished ‘spread’ networks - such as sex work - that enable an STI to enter a population, and ‘maintenance’ networks where each infection leads, on average, to only one new infection [9]. More recently, Blanchard [10], Moses and colleagues [4], and Wilson and Halperin [20] proposed a re-definition of the commonly used terms ‘concentrated’ and ‘generalized’ epidemics. They suggested that instead of classifying epidemics based on HIV prevalence thresholds as we do with the numerical proxy [12], epidemic typologies [4], [20] should reflect the specific behaviours required for HIV to become established in a population - behaviours that must be present for each infection to lead, on average, to more than one new infection [26]. For example, their work suggested that in a ‘concentrated’ epidemic, an effective focused intervention on specific subgroups, such as FSWs, would be sufficient for epidemic control [4], [20]. The objective of the proposed TDEC approach is to classify epidemics as being concentrated, generalizing, or mixed, based on the above work [4], [20]. The framework includes five broad typologies (figures 1 and 2):

- **Concentrated epidemic** [4], [20]: Ongoing transmission requires the presence of a high-risk group (HRG) of individuals within a sexual or needle-sharing network. This HRG may consist of FSWs, high risk men who have sex with men (for example men who sell sex, HR-MSM), IDUs who share needles, or any combination of persons most at risk of HIV transmission. Concentrated epidemics are then divided into three sub-types: i) **local** concentrated epidemics (where the HRG primarily resides in the local region); ii) **non-local** concentrated; and iii) **local and non-local** concentrated epidemics. The distinction between local and non-local depends on whether there is a sufficient local HRG network to sustain ongoing transmission, or whether most local transmission is dependent on “bridging” infections from a high risk network outside the local region (for example, circular male outmigration associated with buying sex at destination [4], [27]). In the prior work by Moses and colleagues, a non-local concentrated epidemic was called a ‘truncated’ epidemic [4]. In all concentrated epidemics, the appropriate HIV prevention policy is to scale up effective focused HIV prevention programmes to reduce transmission in high-risk networks.
- **Generalizing epidemic** [4], [20]: An epidemic is classified as generalizing if the HIV transmission is largely sustained by sexual behaviour in the general population, without any substantial contribution by defined HRGs to overall transmission. In these circumstances, HIV prevention policy should focus on changing sexual behaviour patterns in the general population, particularly focusing on reducing multiple and concurrent partnerships, as well as other prevention technologies, such as, where relevant, increasing the proportion of men who are circumcised.
- **Mixed epidemic** [20]: In mixed epidemics, there is substantial contribution from both the general population sexual behavioural patterns and defined HRGs to overall transmission. In such circumstances, halting and reversing the epidemic depends on a dual strategy of changing sexual behaviour patterns in the general population and reducing transmission in key populations. The relative contribution of the general and key populations varies, and HIV prevention priorities would vary accordingly.

To classify epidemics, we proposed a simple set of decision rules that are based on basic information about the population sexual structure, with the following logic (Table S2):

- Wherever an HIV epidemic occurs and there is a sizable network comprised of a defined HRG, that network will contribute to the overall transmission dynamics, and the epidemic is therefore either concentrated or mixed.
- A generalizing or mixed epidemic only occurs when there is a substantial proportion of the population with multiple and concurrent sexual partnerships, to the extent that HIV transmission could be sustained by these behavioural patterns in the general population.

The crucial and most mutable decision point rests on the differentiation between concentrated and mixed/generalizing epidemic types, since it depends on a judgement as to whether the sexual structure will support HIV transmission in the population apart from identifiable key populations. Conceptually, one approach is to quantitatively assess the extent to which HIV transmission within a population is attributable to sexual partnerships with members of definable key populations [28], [29]. We applied this concept to the appraisal of epidemics in two Indian districts (Belgaum and Bagalkot, since they had the requisite data for the calculation), by applying the standard epidemiological measure of population attributable risk (PAR) to estimate the proportion of prevalent HIV infections among men that could be attributed to sexual partnership with an FSW (Text S1) [28], [29]. In terms of epidemic classification, a very high PAR for male clients would be consistent with a concentrated epidemic, since it would indicate that a large proportion of infections among men are attributable to sexual partnerships with sex workers, and conversely, that very little is attributable to sexual contacts with other types of partners.

The proposed decision tree is one example of how we could try to classify epidemics according to epidemiologic drivers, and was constructed to use available information on each behaviour that could enable sustained HIV transmission. At present, the decision tree is qualitative, but is applied in this study to underscore the importance of and potential for identifying epidemiologic drivers using epidemic appraisals.

## Results

### National-level epidemic appraisals

Table 1 illustrates the findings from the numerical proxy method and the MOT synthesis method at the national level for six countries. With HIV prevalence in the general population exceeding 1%, four of the countries are classified as ‘generalized’ epidemics based on the numerical proxy, with only Peru and India being classified as having ‘concentrated’ epidemics. Following the policy guidance suggested for the numerical proxy classification, the HIV prevention policy in Peru and India should therefore emphasize targeted interventions for key populations, whereas the other four countries should focus on interventions to change behavioural patterns in the general population.

**Table 1:** Results of epidemic appraisals for six countries using the numerical proxy classification and Modes of Transmission (MOT) analysis.

Country	Uganda	Kenya	Nigeria	Peru	Thailand	India
**Numerical proxy classification**	Generalized	Generalized	Generalized	Concentrated	Generalized	Concentrated
HIV prevalence in general population (year) [3]	6.5 (2009)	6.3 (2009)	3.6 (2009)	0.4 (2009)	1.3 (2009)	0.3 (2009)
HIV prevalence within a key population (year)	FSW(47.2;2003)	FSW(31.0;2005)HR-MSM(38.0;2005)IDU(35.0;2004)	FSW(32.7;2007)HR-MSM(13.5;2007)IDU(5.6; 2007)	FSW(0.5;2008)HR-MSM(10.8;2008)	FSW(2.8;2009)HR-MSM(13.5;2009)IDU(38.7;2009)	FSW(4.7;2008)HR-MSM(7.3;2008)IDU(9.2;2008)
**MOT analysis : year**	2008	2008	2009	2010	2005	2010
**Distribution of incident infections (% of total), by subgroup**						
Low-risk heterosexual	42.9	12.3	42.3	16.0	43.4	62.9
Medical injection	0.06	2.2	1.2	0.23	0.8	0.3
Blood transfusion	0.00	0.3	0.5	0.0	0.0	0.0
IDU	0.28	3.8	9.0	1.9	5.7	14.1
Partners of IDU	0.01	0.6	0.4	0.22	1.0	0.55
Female sex workers	0.91	6.6	3.4	0.89	3.9	2.2
Clients	7.8	7.5	4.8	1.3	6.1	7.0
Partners of clients	1.8	1.6	3.4	6.4	8.4	3.0
HR-MSM	0.6	15.2	10.3	54.9	20.9	6.2
Female partners of HR-MSM	0.1	1.3	0.9	6.2	2.5	0.26
Multiple partners (MP)	23.7	20.3	9.1	6.3	3.4	1.2
Partners of MP	21.8	28.3	14.8	5.5	3.9	2.2

HR-MSM (high-risk men who have sex with men); IDU (injecting drug user); FSW (female sex worker). Results were obtained from published sources for the following countries: Uganda (Uganda MOT and Country Report) [17], [21], Kenya (Kenya MOT and Country Report) [5], [22], Nigeria (Nigeria Country Report) [24], Peru (Peru MOT) [25], Thailand [13]. National HIV prevalence estimates for key populations in India were obtained from country level reports [48], [49].

Appraisals using the MOT methodology generated different conclusions than the numerical proxy method, particularly when considering HIV prevention policy. The MOT results indicated that the largest contribution to HIV incidence was among low risk heterosexual populations for four of the countries: Uganda (42.9% of new infections), Nigeria (42.3%), Thailand (43.4%) and India (62.9%). Whereas India is classified as having a ‘concentrated’ epidemic based on the numerical proxy method, the MOT approach indicates that most new infections will occur in the low-risk (general) population, thereby generating contradictory guidance on where to focus prevention resources. HIV prevention policies driven by the MOT method would put minimal priority on FSWs and clients in both Thailand and India, since the overall contribution of these groups (including partners of clients) to new HIV infections works out to approximately 18% in Thailand and 12% in India. Despite much higher HIV prevalence in the general population, the MOT analysis estimates a smaller contribution of new infections in the low risk heterosexual population in Kenya (12.3%) than in Thailand (16.0%).

### District level epidemic appraisals

Results of the numerical proxy, MOT, and TDEC approaches (decision algorithm and PAR in male clients) are presented for six districts of India in Table 2. The numerical proxy method indicates that four of the districts (Shimoga, Belgaum, Bagalkot, and Ganjam) have a ‘generalized’ epidemic. The remaining two districts are classified as ‘low level’. In contrast, the MOT method attributed a high proportion of incident infections to the low-risk heterosexual group in four of the districts (Shimoga, Ganjam, Sikar, and Varanasi; ranging from 73.0 to 89.6% of infections). In Belgaum and Bagalkot, most incident infections were attributed to partners of clients and low-risk groups. The available data for the three north Indian districts (Ganjam, Sikar, and Varanasi) created similar distributions of incident infections by the MOT. There were considerably fewer FSWs per capita in Ganjam, Sikar, and Varanasi as compared with the other districts (Table S2). Notably, each district witnesses high rates of out-migration among its male population, a large proportion of which is seasonal and circular in nature. Anecdotal and limited quantitative evidence from each of these regions suggests that paid sex at their main destination sites is common among out-migrants (Table S2).

**Table 2:** Results of epidemic appraisals for six districts of India using the numerical proxy classification, Modes of Transmission (MOT) analysis and transmission dynamics epidemic classification approach.

District	Shimoga	Belgaum	Bagalkot	Ganjam	Sikar	Varanasi
**Numerical proxy classification**	Generalized	Generalized	Generalized	Generalized	Low level	Low level
HIV prevalence in general population (year, source)	1.0(2008, PPTC)	1.43(2007, GPS)	2.6(2009,GPS)	3.25(2008,ANC)	0.98(2004,GPS)	0.25(2008,PPTC)
HIV prevalence within a core-group (year)	FSW (9.0;2008)HR-MSM (9.9; 2008)	FSW (27.3; 2008)HR-MSM (10.6; 2008)	FSW (5.3; 2008)HR-MSM (13.0; 2008)	N/A	N/A	FSW (0.8;2006)
**MOT analysis: year**	2010	2010	2010	2010	2010	2010
**Distribution of incident infections (% of total), by subgroup**						
Low-risk heterosexual	73.0	12.5	35.8	87.1	89.6	81.1
Medical injection	0.52	1.3	1.3	1.2	1.2	1.1
Blood transfusion	0.0	0.0	0.0	0.0	0.0	0.0
IDU	0.0	2.9	0.0	0.0	0.44	5.9
Partners of IDU	0.0	1.8	0.0	0.0	0.01	0.2
FSW	0.76	1.6	7.8	0.03	0.09	0.34
Clients	2.5	17.6	4.5	0.02	0.06	0.12
Partners of clients	18.2	37.3	47.1	0.27	1.1	2.5
HR-MSM	2.8	3.8	1.7	0.0	0.0	1.8
Female partners of HR-MSM	0.35	0.62	1.2	0.0	0.0	0.11
Multiple partners(MP)	0.73	5.9	0.36	6.5	3.4	3.2
Partners of MP	1.2	14.6	0.25	4.8	4.0	3.6
**Transmission Dynamic Epidemic Classification**						
**Decision Algorithm**	Concentrated - local	Concentrated (local & non-local) or mixed	Concentrated (local & non-local) or mixed	Concentrated (local & non-local)	Concentrated (local & non-local) or mixed	Concentrated (local & non-local)
**PAF (male clients)**	N/A	76.1%	90.1%	N/A	N/A	N/A

ANC (Antenatal clinic [sentinel surveillance]); PPTC(prevention of parent to child transmission clinic); GPS (general population survey); FSW (female sex worker); HR-MSM (high-risk men who have sex with men); IDU (injecting drug user); PAF (population attributable fraction); N/A (data not available).

Classification of regional epidemics using the alternate decision algorithm indicated that all six districts were entirely or predominantly concentrated. In three of the districts (Belgaum, Bagalkot and Sikar), a low to moderate level of multiple partnerships in the general population suggested that these epidemics might also be considered mixed, but with key populations playing a dominant role in the transmission dynamics. However, the PAR analysis in Belgaum and Bagalkot confirmed that these epidemics are predominantly concentrated, with 76.1% and 90.1% of prevalent HIV infections among men attributable to being a client of a female sex worker.

### Sensitivity analysis of the MOT method

The predicted distribution of incident infections was dependent on assumptions of relative population size and model structure. Total adult population is fixed such that the fractional size of the low-risk group is dependent on the size of other groups. Reducing the size of the low-risk group as a result of increasing the size of another subgroup altered the findings considerably. For example, 6% of adult males in the Peruvian MOT [25] were assumed to be HR-MSM as compared with 0.1% in India. Application of the Peruvian estimate of HR-MSM population size along with joint estimates of 2.47 partnerships/year and 11.6 sex acts/partnership [25] for HR-MSM in India, while all other Indian parameters were held constant, produced a redistribution of incident infections: approximately 61% of incident HIV infections were now acquired among Indian HR-MSM.

**Figure 3:**
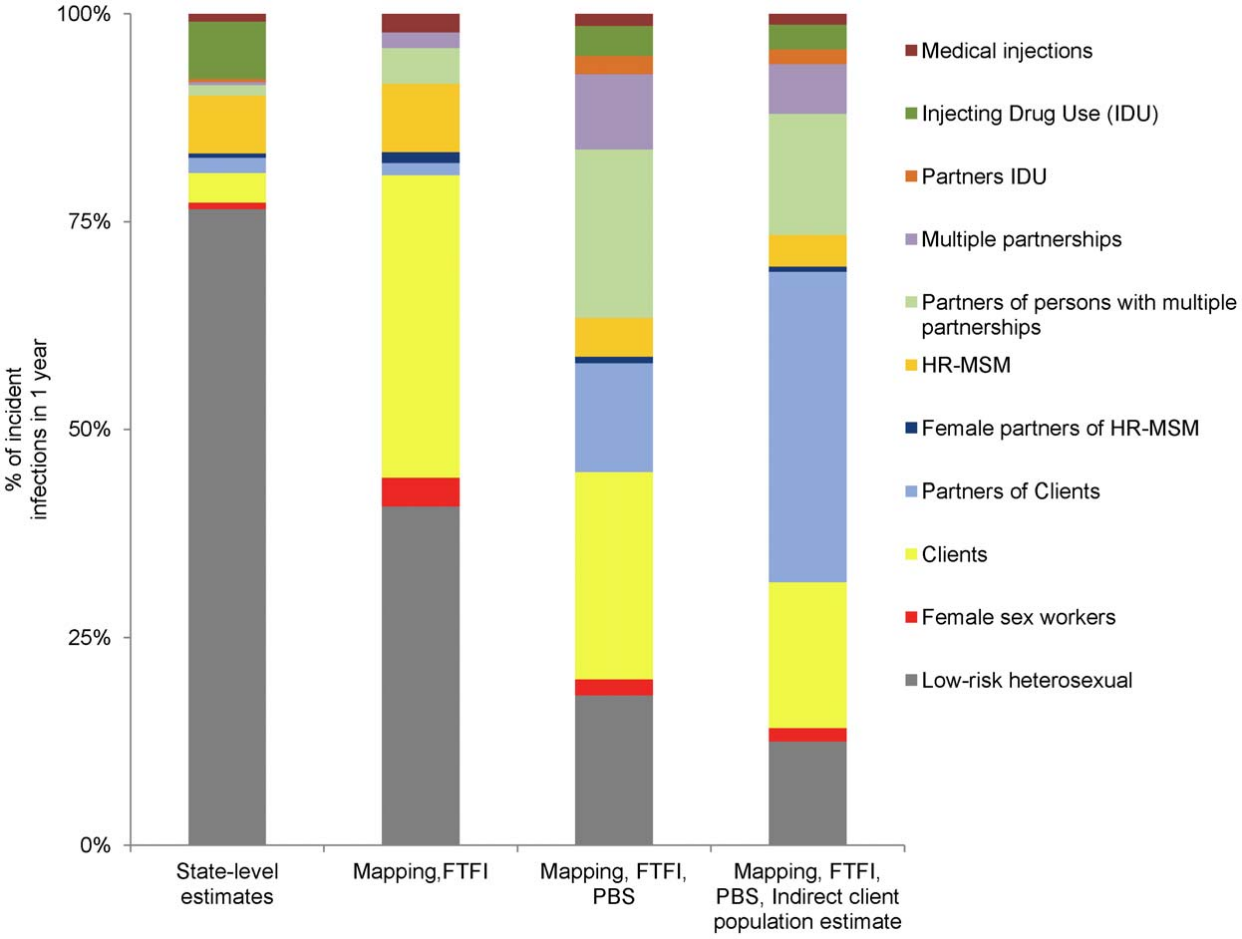
Modes of Transmission sensitivity analysis with various tiers of data-sources for Belgaum, India. FTFI, face to face interview; HRG, high-risk group; PBS, polling booth survey. Indirect client estimate was based on reported client volume by rural and urban female sex workers. Redistribution in incident infections is driven predominantly by the estimate of the client population (3.0 to 16.8%) and multiple partnerships (3.1 and 0.8% among males and females respectively using state-level estimates and 9.9 and 1.5% among males and females respectively using PBS estimates). Increasing the population size of each of these risk groups resulted in a predominance of infections among direct partners of clients and persons engaged in multiple partnerships.

Figure 3 shows how the MOT results would vary for one district in India (Belgaum) depending on the source of population size estimates for clients of FSWs. If state-level estimates based on self-reported client status are used, 5.4% of HIV incidence is attributed to clients and their partners, with over 75% of HIV incidence attributed to the low risk heterosexual group. Behavioural surveys conducted within the district yielded higher estimates of the size of the male client population and the proportion of infections associated with clients, but differed depending on whether the survey methodology included face-to-face interviews or more anonymous data collection through a polling booth survey. Estimates of the size of the client population and their contribution to HIV incidence were much higher when an indirect method was used based on the mapped size of the FSW population, their client volume, and data on the frequency of visits by clients, with over 70% of incident cases being attributed to FSWs, clients and other sexual partners of clients.

## Discussion

Epidemic appraisals that reliably and accurately reflect underlying transmission dynamics are central to the development of effective HIV prevention strategies and resource allocation [20]. Thus far, two methods have been developed and used widely for this purpose, and as illustrated in this paper, they can provide differing assessments of HIV epidemics and thereby contradictory guidance for HIV prevention policy. The arbitrary thresholds of the numerical proxy approach frequently misclassify concentrated and mixed epidemics as ‘generalized’, thereby implicitly downplaying the relative importance of HIV prevention on key populations [30]. As we illustrate here, Thailand and four out of six districts in India would be classified as ‘generalized’ epidemics based on the numerical proxy, despite substantial evidence that these epidemics are better classified as concentrated with respect to their transmission dynamics and HIV prevention strategies [4], [31], [32], [33]. Our findings highlight the lack of a robust empirical basis for the prevalence thresholds used in the numerical proxy. And while its influence on directing HIV prevention strategies is waning [20], the approach remains prominent in the discourse around HIV prevention policy [34].

The MOT model was developed to address the shortcomings of the numerical proxy by quantifying incidence in order to provide better guidance for HIV prevention strategies [16]. However, as we have illustrated, there are important limitations with this approach. First, by focusing on the distribution of new HIV infections over a very short and current time period (i.e. the subsequent one year), the MOT model is prone to mis-specifying the underlying transmission dynamics and thereby misguiding HIV prevention policy. Even in the few districts (Belgaum and Bagalkot) where most infections were distributed among partners of clients and a reasonable interpretation would attribute these infections to sex work at a proximal point in time, the MOT results could also be translated erroneously to prioritize prevention efforts on partners of clients rather than FSWs. It is important to note that the latter interpretation results from the conceptual framework and methodology itself, not from inaccurate parameter inputs with respect to the population sexual structure. By focusing only on the distribution of new infections in the coming year, the appraisal fails to capture the medium and longer term transmission dynamics because it inherently ignores the more proximate transmission pathways. Moreover, by focusing on the HIV prevalence distribution according to current behavioural characteristics, it fails to consider behavioural changes within individuals over time. From polling booth surveys in south India, 4.6% of adult males reported they had sex with an FSW in the last year, while 10.9% reported they had sex with an FSW at some time in their past [23]. Therefore, individuals who are currently in a low-risk category may have been infected previously when they were part of high risk sexual networks [35]. So, with temporal behaviour changes at the individual level and the stereotypical propagation of the epidemic from higher risk networks to lower risk partnerships [9], as an HIV epidemic progresses over time there will be an increasing shift in the distribution of new infections from current high-risk populations to low risk partnerships [36]. Our results show that the MOT model tends to amplify this shift to “downstream” infections in highly concentrated epidemics, since the relative size of the low risk heterosexual population is much larger in those contexts than in more generalized epidemics. With respect to HIV prevention policy, epidemic appraisals using the MOT model will tend to place excessive emphasis on strategies that prevent infections at the terminus of transmission chains, thereby reducing the population level impact. In concentrated epidemics, this would entail inappropriately de-emphasizing interventions for key populations. In generalized epidemics, greater focus might be placed on discordant couples who are currently monogamous, rather than focusing on reducing high-risk sexual behaviour patterns such as multiple and concurrent partnerships, which not only drive the epidemic at a population level, but are also the risk factors for HIV acquisition by individuals before they enter into monogamous sexual partnerships.

As demonstrated in our case study of India, a second constraint of the MOT approach is that it is difficult to apply the method at a geographic or jurisdictional level that captures the epidemic heterogeneity found within many countries. In large countries with heterogeneous epidemics such as India and Nigeria, national level MOT analyses are of limited value, since HIV prevention plans are increasingly decentralized to the state, district, or local level. Recognizing this heterogeneity, some countries are now conducting MOT analyses at the sub-national level, but often do so without the requisite local data to parameterize the model. As a result, data are borrowed from national studies or studies in other local jurisdictions [18], thereby negating the value of local level MOT analyses. One solution involves extending the necessary data collection to the state and local levels. However, even if resources were available to do so, it is important to note a third issue with the MOT; its high sensitivity to variations in parameters describing the population sexual structure.

Our analysis of Belgaum district in India, which is rich in the data required for MOT estimates, illustrates that variations in a parameter such as population size of clients, depending on data sources, greatly influences MOT method results. Of note, the source often used to parameterize MOT models [21], [22], [37], a population-based survey of self-reported behaviour, resulted in lower estimates of the contribution of FSWs and clients to the HIV incidence due to lower estimates of the size of the client population. We believe that there is considerable merit in an indirect approach to estimating the size of the client population. We used estimates of the size of the FSW population, which have been substantially verified through programme registration data, along with an estimation of the average number of different client partners for FSWs within a year, and believe that this is a fairly robust method that could be applied more widely.

Beyond constraints with epidemic appraisal methodologies *per se*, with a growing number of countries conducting such appraisals, inconsistencies are emerging in how the results are interpreted and translated into policy. For example, the most recent UNAIDS report from Kenya acknowledged that despite a paucity of quantitative data on high-risk groups, there was evidence to suggest that interventions targeted at HRGs are urgently needed, and described its epidemic as mixed [5]. In Malawi, researchers have utilized the MOT model to identify prevention targets. Despite an FSW population size of 1.58% and HIV prevalence of 70.7%, alongside an estimated client population of 17.7% with an HIV prevalence of 17%, the majority of incident infections were predicted to occur among low-risk individuals (36%) [18]. As a result, 100% condom use among serodiscordant couples was identified as the highest priority single intervention [18]. The contrasting examples of Kenya and Malawi highlight the variability in interpretation and consequently, misinterpretation, of the MOT results. Because most infections were predicted to occur among individuals reporting one partner in the preceding year, followed by individuals reporting more than one partner, the MOT synthesis in Lesotho prioritized ‘steady couples’ and extra-marital sex as the focus for prevention [37]. Indeed, most of the MOT-based policy documents in Africa prioritize interventions aimed at the low-risk populations [19], [21], [24], [37], and relative funding and prevention programmes for key populations in this region remains low to negligible [38]. These examples suggest that rather than leading to a more consistent approach to translating epidemic appraisal into HIV prevention policy, strategy development may actually have become more haphazard.

To address the above limitations, we have proposed and applied an example of an alternative approach to characterizing local HIV epidemics, based on previous work on how HIV epidemics could be characterized [4], [9], [10], [20]. This appraisal aims to identify drivers of the epidemic, so that prevention programmes can focus on the proximal source of infections to achieve a long-term impact. Although this appraisal approach benefits from detailed data (for example, to calculate the PAR of paid sex on male HIV infections), it could be also be conducted with information gathered by key informant and stakeholder interviews. Importantly, the method requires that we view the epidemiology of HIV through the lens of transmission pathways. We developed this appraisal as a simple explanatory framework with a goal to provide broad insights for local HIV prevention strategies. The proposed framework has been assessed against empirical data from India. We recognize that in order to be useful, the algorithm requires validation and refinement against empirical data from other regions of the world as well as testing and refinement with simulated epidemics. Important refinements include quantifying thresholds at each point in the decision tree, including migration and how migration may or may not contribute to local HIV transmission [39], [40], and we are currently pursuing this using simulated HIV epidemics. Further empirical work on assessing thresholds will also be needed before such an algorithm can be widely applied. Despite these limitations, the qualitative decision tree demonstrates that there exists the potential for classifying epidemics on the basis of epidemic drivers.

In the context of HIV prevention, the purpose of the current and proposed epidemic appraisals is to broadly direct a prevention strategy [20]. Having laid out an overall strategy, programmes still need to understand the underlying structural factors along the pathway to HIV vulnerability [41], including gender inequality, income disparity, non-injecting substance abuse and dependence, or sexual violence, in order to address the structural component of a combination prevention approach [41], [42]. We also recognize that in addition to appraisals that focus on subgroups and their role in transmission dynamics, methods need to be developed that explicitly characterize place and settings [43], [44], particularly in settings where spatially complex and diverse STI transmission dynamics are underway. Identifying epidemiologic drivers requires regional information from key informants or mapping of HRGs [45], data that is important for place- or setting-based HIV prevention programmes. A setting-based approach necessarily makes use of information on epidemiologic drivers, so as not to miss locales with hidden HRGs [46]. When appraisals like the MOT fail to identify the key behaviours that enable ongoing HIV transmission at a population-level, particularly in concentrated and mixed epidemics, they can divert the prevention focus from HRGs who are often socially marginalized yet incur the largest per-capita incidence of HIV. In light of the potential bias demonstrated with the MOT, HIV prevention for reasons of social justice could play a larger role in ensuring resources are focused on HRGs. Hence, HIV epidemic appraisals constitute one of many sources of evidence and perspectives that policy makers utilize when designing prevention programmes [47].

In conclusion, the arbitrary thresholds imposed by the numerical proxy method and the use of poor inputs in the MOT method limit the validity of their respective results. Accurate parameters could increase the reliability of MOT predictions, and the importance of accurately quantifying the distribution of incident infections in one year for the provision of testing and treatment calls for improved parameterization of the MOT method and its ongoing use. However, these traditional appraisals remain restricted to a snapshot of prevalent and incident HIV infections in a region and discount individual dynamism and epidemic trajectory. To reliably impact on the trajectory of the HIV epidemic, we must shift from using one-year metrics in isolation to examining epidemiologic drivers when we design prevention programmes. And we must do so before our short-term priorities potentially lead to the misuse of resources in the name of long-term prevention.

## Supporting Information

Text S1 **Data sources for the numerical proxy, Modes of Transmission, and transmission dynamics epidemic classification approaches to HIV epidemics in India and 6 Indian districts.**
(DOC)

Table S1 **Input values for the Modes of Transmission analysis.**
(DOC)

Table S2 **Transmission dynamics epidemic classification.**
(DOC)
