# Supplementary material for: HIV Epidemic Appraisals for Assisting in the Design of Effective Prevention Programmes: Shifting the Paradigm Back to Basics
Source: PLoS One. 2012 Mar 1;7(3):e32324. doi: 10.1371/journal.pone.0032324 (PMC3291609; doi:10.1371/journal.pone.0032324)
Supplement: Text S1 — Data sources for the numerical proxy, Modes of Transmission, and transmission dynamics epidemic classification approaches to HIV epidemics in India and 6 Indian districts. (DOC) [file pone.0032324.s001.doc]

**Text S1**

Data sources for the numerical proxy, Modes of Transmission, and Transmission Dynamics Epidemic Classification (TDEC) approaches to HIV epidemics in India and 6 Indian districts.

Data sources

*Demographic data*

Demographic data came primarily from publicly available 2001 India Census Data [1] projected to 2006. Data for subgroup population size estimates were obtained from local non-governmental organization (NGO) mapping[2,3,4,5,6,7,8,9,10,11] and the Karnataka Health Promotion Trust [10] as well as general population surveys [12,13]. The indirect client population size estimate was obtained as follows:


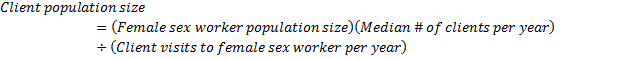


The median client volume per year was obtained from the female sex worker (FSW) Integrated Biological and Behavioural Assessments (IBBA) or NGO registration data[2,3,4,5,6,7,9,10,11], and client visits to FSWs was obtained from the client IBBA [14,15,16,17,18,19,20].

*HIV prevalence data*

HIV prevalence data came primarily from India’s HIV sentinel surveillance system [21]. Where possible, the most recent parameters were used. To generate HIV estimates for high risk populations, sentinel surveillance data, behavioural surveillance surveys (IBBA, polling booth surveys [PBS], and general population surveys [GPS]), and NGO registration data were used [10,12,13,18,19,20,22,23,24,25,26,27,28]. To obtain HIV prevalence for the remainder of the population (e.g. low-risk individuals and regular sex partners of high-risk individuals) we used the overall HIV prevalence in the region from GPS when available, and from antenatal clinic sentinel surveillance otherwise [29,30,31].

*Missing data*

Missing HIV prevalence and population size data were imputed using data from the broader geographical region; for example, state-level estimates were used when district-level estimates were not available [21,23,25,33,32].

Estimation methods

Estimates of incident HIV infections were generated using the Workbook Method recommended by the UNAIDS and WHO for the Modes of Transmission (MOT) assessment [33,34,35].

To estimate the population attributable fraction (PAR) of paid sex for HIV infections among males, we required data on the HIV prevalence risk ratio or relative risk (RR) of HIV for male clients compared to non-clients and the estimated proportion of the adult male population that were clients to calculate the PAR (%) using the formula:[36,37,38]


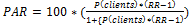
 …(1)

Where P(clients) refers to the fraction of the total male population that has paid for sex, and the RR is given by:


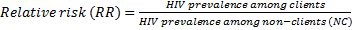
 …(2)

References

1. Government of India Ministry of Home Affairs (2001) Census India. Registrar General & Census Commissioner, India.

2. ARUNA (2006) Annual progress reports: Ganjam. Bhubaneshwar: Association for Rural Uplift and National Allegiance.

3. Choudhury S (2010) Migration and vulnerability of HIV/AIDS in Ganjam, India. Bangalore: Karnataka Health Promotion Trust.

4. GPSS (2007) Needs assessment survey of Digapahandi Block. Ganjam: Govinda Pradhan Smruti

Sansad.

5. GPSS (2008) Needs assessment survey of in-migrants in Ganjam district, India. Ganjam: Govinda

Pradhan Smruti Sansad.

6. GPSS and ARUNA (2010) Migration in Ganjam. Ganjam: Association for Rural Uplift and National

Allegiance and the Govinda Pradhan Smruti Sansad.

7. LEPRA (2004) Baseline survey of Aska and Hinjilikatu block. Ganjam: LEPRA: Health In Action.

8. Government of Orissa (2007) District statistical handbook of Ganjam. Bhubaneshwar: Directorate of

Economics and Statistics.

9. India Health Action Trust (2010) HIV/AIDS situation and response in Uttar Pradesh: Epidemiological

appraisal using data triangulation. Bangalore.

10. India Health Action Trust (2010) HIV/AIDS situation and response in Karnataka: Epidemiological

appraisal using data triangulation. Bangalore.

11. Raman Development Consultants (2009) Mapping of high risk groups and migratns in Rajasthan,

2009. Jaipur.

12. Rajaram S, Sangameshwar S, Jayachandran A, Bradley J, Alary M, et al. (2008) HIV and STIs in

Belgaum district, Karnataka, India. A general population survey. Bangalore. Available:

<http://www.khpt.org/charme.html>. Accessed 2 October 2011.

13. Rajaram S, Bradley J, Alary M, Ramesh B, Washington R, et al. (2010) HIV and STIs in Bagalkot

district, Karnataka, India. A general population survey. Bangalore. Available:

http://www.khpt.org/charme.html. Accessed 2 October 2011.

14. Ramesh BM, Beattie TSH, Shajy I, Washington R, Jagannathan L, et al. (2010) Changes in risk

behaviours and prevalence of sexually transmitted infections following HIV preventive interventions

among female sex workers in five districts in Karnataka state, south India. Sex Transm

Infect 86: I17-I24.

15. Saidel T, Adhikary R, Mainkar M, Dale J, Loo V, et al. (2008) Baseline integrated behavioural and

biological assessment among most at-risk populations in six high-prevalence states of India: design and

implementation challenges. AIDS 22: S17-S34.

16. Reza-Paul S, Beattie T, Syed HUR, Venukumar KT, Venugopal MS, et al. (2008) Declines in risk

behaviour and sexually transmitted infection prevalence following a community-led HIV preventive

intervention among female sex workers in Mysore, India. AIDS 22: S91-S100.

17. Karnataka Health Promotion Trust (2006) Sampling and design: Integrated behavioral and biological

assessment in Karnataka.

18. Karnataka Health Promotion Trust (2008) Integrated Biological & Behavioral Assessment: 2005

(round 1) and 2008 (round 2).

19. Karnataka Health Promotion Trust (2008) Female sex workers IBBA chapter report: Belgaum.

Bangalore, India.

20. Karnataka Health Promotion Trust (2008) Female sex wokers IBBA chapter report: Shimoga.

Bangalore, India.

21. National AIDS Control Organization (2010) UNAIDS Country Progress Report: India. Available:

<http://www.unaids.org/en/dataanalysis/monitoringcountryprogress/2010progressreportssubmittedbycountr>

ies/ . Accessed 2 October 2011.

22. Indian Council of Medical Research (2007) India HIV estimates 2006: technical report. National

AIDS Control Organization. Available:

http://www.nacoonline.org/Quick_Links/Publication/ME_and_Research_Surveillance/ . Accessed 2

October 2011.

23. Ministry of health and family welfare National behavioural surveillance survey 2006: female sex

workers and their clients. Delhi: National AIDS Control Organization, Government of India. Available:

<http://www.nacoonline.org/Quick_Links/Publication/ME_and_Research_Surveillance/>. Accessed 2

October 2011.

24. Ministry of health and family welfare National behavioural surveillance survey 2006: men who have

sex with men and injecting drug users. Delhi: National AIDS Control Organization, Government of India.

Available: http://www.nacoonline.org/Quick_Links/Publication/ME_and_Research_Surveillance/ .

Accessed 2 October 2011.

25. Ministry of health and family welfare National behavioural surveillance survey 2006: general

population. Delhi: National AIDS Control Organization, Government of India. Available:

http://www.nacoonline.org/Quick_Links/Publication/ME_and_Research_Surveillance/ . Accessed 2

October 2011.

26. Gelmon L, Singh K, Singh P, Costigan A (2006) The sexual networking study from the out-migrant

community of the Shekawati region of Rajasthan: 2004-2005. Jaipur, India.

27. Brahmam GNV, Kodavallaa V, Rajkumar H, Rachakulla HK, Kallam S, et al. (2008) Sexual

practices, HIV and sexually transmitted infections among self-identified men who have sex with men in

four high HIV prevalence states of India. AIDS 22: S45-S57.

28. Karnataka Health Promotion Trust (2007) Polling booth survey findings. KHPT data dissemination

workshop. Bangalore.

29. National AIDS Control Organization (2009) Annual report 2008-2009. New Delhi: Ministry of Health

& Family Welfare, Government of India. Available:

http://www.nacoonline.org/Quick_Links/Publication/ME_and_Research_Surveillance/ . Accessed 2

October 2011.

30. National AIDS Control Organization (2009) Mid-term review of NACP-III. Delhi: Government of

India. Available: http://www.nacoonline.org/Quick_Links/Publication/ME_and_Research_Surveillance/ .

Accessed 2 October 2011.

31. National AIDS Control organization (2010) Press release: HIV declining in India. Delhi: Ministry of

health and family welfare, Government of India. Available:

http://www.nacoonline.org/Quick_Links/Publication/ME_and_Research_Surveillance/ . Accessed 2

October 2011.

32. National AIDS Control Organization. Sentinel surveillance among high-risk groups in Karnataka;

2008; Bangalore, India. Karnataka Health Promotion Trust.

33. Lyerla R, Gouws E, Garcia-Calleja JM, Zaniewski E (2006) The 2005 Workbook: an improved tool

for estimating HIV prevalence in countries with low level and concentrated epidemics. Sex

Transm Infect 82 Suppl 3: 41-44.

34. Gouws E, White PJ, Stover J, Brown T (2006) Short term estimates of adult HIV incidence by mode

of transmission: Kenya and Thailand as examples. Sex Transm Infect 82 Suppl 3: 51-55.

35. UNAIDS (2007) Modelling the expected short-term distribution of incidence of HIV infections by

exposure group. Geneva. Available:

http://www.unaids.org/en/dataanalysis/tools/incidencebymodesoftransmission/ . Accessed 2 October

2011.

36. Alary M, Lowndes CM (2004) The central role of clients of female sex workers in the dynamics of

heterosexual HIV transmission in sub-Saharan Africa. AIDS 18: 945-947.

37. Lowndes CM, Alary M, Meda H, Gnintoungbe CAB, Mukenge-Tshibaka L, et al. (2002) Role of core

and bridging groups in the transmission dynamics of HIV and STIs in Cotonu, Benin, West Africa.

Sex Transm Infect 78 Suppl 1: 69-77.

38. Cote AM, Sobela F, Dzokoto A, Nzambi K, Asamoah-Adu C, et al. (2004) Transactional sex is the

driving force in the dynamics of HIV in Accra, Ghana. AIDS 18: 917-925.
