# Supplementary material for: HIV Epidemic Appraisals for Assisting in the Design of Effective Prevention Programmes: Shifting the Paradigm Back to Basics
Source: PLoS One. 2012 Mar 1;7(3):e32324. doi: 10.1371/journal.pone.0032324 (PMC3291609; doi:10.1371/journal.pone.0032324)
Supplement: Table S1 — Input values for the Modes of Transmission analysis. (DOC) [file pone.0032324.s002.doc]

**Table S1: Input values for Modes of Transmission Analysis**

**Table S1a**. Input values, sources, and assumptions for the Modes of Transmission Analysis for India.

| **Input descriptor** | **Default value (range)** | **Source(s)** | **Notes** |
| --- | --- | --- | --- |
| **IDU**  Population size (% of adult female pop.)  HIV prevalence (%)  STI prevalence (%)  # of partners / year  # of sex acts / partner / year  % of acts protected | 0.06  9.2  11.9  90  2  50 | UNAIDS Country Progress Report 2010  Sentinel surveillance  BSS 2006 (IDU)  BSS 2006 (IDU)  Assumption  BSS 2006 (IDU) | BSS for IDU population restricted to 10 cities across India.  # of partners/year based on median frequency of sharing /using needle previously used by someone else (occasionally per month – assumed to be 7⋅.5 injections / month)  % of acts protected based on median frequency of cleaning used needle prior to use (half the time per month) |
| **Partners of IDU**  Population size (% of adult female pop.)  HIV prevalence (%)  STI prevalence (%)  # of partners / year  # of sex acts / partner / year  % of acts protected | 0.03  0.3  N/A  1  52  19.5 | BSS 2006 (IDU)  Assumption (overall prevalence)  Belgaum GPS  BSS 2006 (General Population) | Population size based on % of married men living with spouse, married men living with other sexual partner, unmarried men living with a sexual partner. Sexual partner in each case assumed to be female.  Number of sex acts in a married or cohabitating relationship estimated from median # frequency of sex reported by men and women in the Belgaum GPS  Consistent condom use reported by females with spouse/regular partner in the last 12 months (19.5) |
| **FSW**  Population size (% of adult female pop.)  HIV prevalence (%)  STI prevalence (%)  # of partners / year  # of sex acts / partner / year  % of acts protected | 0.45  4.9  25.2  504  1  73.4 | UNAIDS Country Progress Report 2010  Sentinel Surveillance  BSS 2006 (FSW)  BSS 2006 (FSW)  No data source  BSS 2006 (FSW) | Assumed prevalence of GUD was 50% of all cases of symptomatic STIs in last 1 year  % of acts protected based on consistent condom use with clients in the last 3 months. |
| **Clients of FSWs**  Population size (% of adult male pop.)  HIV prevalence (%)  STI prevalence (%)  # of partners / year  # of sex acts / partner / year  % of acts protected | 2.4  0.96  12  4.4  15  74 | BSS 2006 (General population)  NFHS-3  BSS 2006 (Clients)  BSS 2006 (Clients)  Assumption, no data  BSS 2006 (Clients) | Client population based on % of men reporting having paid for sex in last 1 year (BSS 2006)  # of sex acts/partner/year approximated to almost equal total # of FSW sex acts / year  % of acts protected based on consistent condom use with FSWs in the last 3 months. |
| **Female partners of clients**  Population size (% of adult female pop.)  HIV prevalence (%)  STI prevalence (%)  # of partners / year  # of sex acts / partner / year  % of acts protected | 1.39  0.3  N/A  1  52  19.5 | BSS 2006 (Clients)  UNAIDS country assessment  Assumption  Belgaum GPS  BSS 2006 (General Population) | Population size estimation based on % of men reporting currently married & living with spouse and unmarried men living with female partner |
| **HR-MSM**  Population size (% of adult male pop.)  HIV prevalence (%)  STI prevalence (%)  # of partners / year  # of sex acts / partner / year  % of acts protected | 0.12  7.3  3.2  78  1  71.4 | UNAIDS Country Progress Report 2010  Sentinel surveillance  IBBA (MSM, 2006-2007)  BSS 2006 (MSM)  Assumption, no data  BSS 2006 (MSM) | IBBA in 4 states (Karnataka, Andhra Pradesh, Maharastra, Tamil Nadu)  BSS among MSM restricted to 10 cities.  % of sex acts protected based on consistent condom use among HR-MSM in the last 6 months. |
| **Female partners of HR-MSM**  Population size (% of adult female pop.)  HIV prevalence (%)  STI prevalence (%)  # of partners / year  # of sex acts / partner / year  % of acts protected | 0.04  0.3  N/A  1  26  18.2 | BSS 2006 (MSM)  Assumption (overall HIV prevalence)  Assumption  Assumption  BSS 2006 (MSM) | Population size estimation based on % of all MSM who are currently married & living with spouse and unmarried living with female partner.  Assumed # of sex acts with female sex partner approximately half that of non high risk MSM-female relationships. |
| **Multiple partnerships (MP)**  Population size (% of adult male/female pop.)  HIV prevalence (%)  STI prevalence (%)  # of partners / year  # of sex acts / partner / year  % of acts protected | Male (2.04),Female (0.05)  0.84  3.2  2  26  41.8 | NFHS-3  NFHS-3  BSS 2006 (General Population)  NFHS-3  Assumption, no data  BSS 2006 (General Population) | Assumed # of sex acts with casual sex partner approximately half that of married or cohabitating relationship.  Consistent condom use among non-regular partners in the last 12 months (41.8%) |
| **Partners of MP**  Population size estimation  HIV prevalence (%)  STI prevalence (%)  # of partners / year  # of sex acts / partner / year  % of acts protected | Males (0.04),Females(1.31)  0.3  N/A  1  52  16.3 | NFHS-3  Assumption (overall prevalence)  Assumption  Belgaum GPS  BSS 2006 (General Population) | Number of sex acts in a married or cohabitating relationship estimated from median # frequency of sex reported by men and women in the Belgaum GPS  Consistent condom use among spouse/regular partner in the last 12 months (16.3%) |
| **Low-risk heterosexual**  Population size estimation  HIV prevalence (%)  STI prevalence (%)  # of partners / year  # of sex acts / partner / year  % of acts protected | Male (87.8),Female(86.6)  0.3  0  1  52  16.3 | NFHS-3  Assumption (overall prevalence)  Assumption, no data  NFHS-3  Belgaum GPS  BSS 2006 (General Population) | Consistent condom use among spouse/regular partner in the last 12 months (16.3%) |
| **No risk**  Population size estimation  HIV prevalence (%)  STI prevalence (%)  # of partners / year  # of sex acts / partner / year  % of acts protected | Male(7.5),Female(10.0)  0.3  N/A  0  N/A  N/A | NFHS-3  Assumption (overall prevalence) |  |
| **Medical injections**  Population size estimation  HIV prevalence (%)  STI prevalence (%)  # of partners / year  # of sex acts / partner / year  % of acts protected | Male(35.7),Female(38.5)  0.3  N/A  2 (injections/year)  1  95 | NFHS-3  Assumption (overall prevalence)  NFHS-3  Set value  NFHS-3 |  |
| **Blood transfusions**  Population size estimation  HIV prevalence (%)  STI prevalence (%)  # of partners / year  # of sex acts / partner / year  % of acts protected | Male(0.1),Female(0.13)  0.3  N/A  1  1  100 | NFHS-3  Assumption (overall prevalence)  NACO (2004) | # of transfusions per 100 persons per year estimated from NFHS-3 assuming individuals are only transfused 1 in their life. |
| **% of adult males circumcised** | 12.7 | NFHS-3 |  |

ANC (Ante-natal clinic)[1,2,3,4,5]; MP (multiple partnerships); GPS (general population survey[1,6,7]); PBS (polling booth survey[1]); NACO (National AIDS Control Organization); FSW (female sex worker); HR-MSM (high-risk men who have sex with men); STI (sexually transmitted infection); NFHS-3 (National Family Health Survey Round 3[8]); BSS (Behavioural Surveillance Survey[9,10,11]); IBBA (Integrated Biological and Behavioural Assessment[1,12,13,14,15,16,17,18]). UNAIDS Country Progress Report 2010[4]

**Table S1b. Input values, sources, and assumptions for the Modes of Transmission Analysis for Bagalkot district**, India.

| **Input descriptor** | **Default value (range)** | **Source(s)** | **Assumptions** |
| --- | --- | --- | --- |
| **IDU**  Population size (% of adult pop.)  HIV prevalence (%)  STI prevalence (%)  # of shared needles / year  # of shared needles/partner/ year  % of acts protected | 0(Male), 0(Female)  N/A  N/A  N/A  N/A  N/A | GPS 2009; Mapping data |  |
| **Partners of IDU**  Population size (% of adult female pop.)  HIV prevalence (%)  STI prevalence (%)  # of partners / year  # of sex acts / partner / year  % of acts protected | 0(Male), 0(Female)  N/A  N/A  N/A  N/A  N/A |  |  |
| **FSW**  Population size (% of adult female pop.)  HIV prevalence (%)  STI prevalence (%)  # of partners / year  # of sex acts / partner / year  % of acts protected | 1.59  5.3  33.2  374  1  46.1 | Mapping data (2008)  Sentinel surveillance (state-level)  State-level est. BSS 2006  NGO registration data  Assumption | Because the frequency of sex acts with repeat clients was not available, total client volume (with 1 exposure per client) was used.  % of acts protected based on consistent condom use with repeat and occasional clients (weighted average) as reported by clients (IBBA).  % STI prevalence based on 50% of reported STI symptoms. |
| **Clients of FSWs**  Population size (% of adult male pop.)  HIV prevalence (%)  STI prevalence (%)  # of partners / year  # of sex acts / partner / year  % of acts protected | 17.3  13.4  15.0  23  1  46.0 | indirect method  IBBA 2009  IBBA 2009  IBBA 2009  Assumption  IBBA 2007 | # of partners/year includes only FSWs because this estimate is used to approximate whether total # of sex acts/client/year is close to that of FSWs.  # of sex acts/partner/year based on two findings; (1) # of sex acts for FSWs/year has to be near equal to # of sex acts for clients, and (2) median duration from time of last sex with a repeat FSW was 5 days in the cross-sectional IBBA 2007. |
| **Female partners of clients**  Population size (% of adult female pop.)  HIV prevalence (%)  STI prevalence (%)  # of partners / year  # of sex acts / partner / year  % of acts protected | 13.3  1.24  N/A  1  52  4.9 | GPS (PBS,Indirect)  GPS 2009 (married Females)  Assumption  Belgaum GPS  IBBA 2009 | Population size estimation based on % of men reporting currently married & living with spouse and unmarried men living with female partner (IBBA 2008) |
| **HR-MSM**  Population size (% of adult male pop.)  HIV prevalence (%)  STI prevalence (%)  # of partners / year  # of sex acts / partner / year  % of acts protected | 0.32  13.0  3.2  104  1  89.0 | Mapping data (2008)  Sentinel (state-level est.)  National level est.  NGO registration data  Assumption  PBS 2008 |  |
| **Female partners of HR-MSM**  Population size (% of adult female pop.)  HIV prevalence (%)  STI prevalence (%)  # of partners / year  # of sex acts / partner / year  % of acts protected | 0.23  1.24  N/A  1  120  16.7 | GPS 2009 (Married females)  Assumption  Belgaum estimate  PBS 2008 | Population size estimation based on % of all men who are currently married & living with spouse and unmarried living with female partner (GPS 2009). |
| **Multiple partnerships (MP)**  Population size (% of adult pop.)  HIV prevalence (%)  STI prevalence (%)  # of partners / year  # of sex acts / partner / year  % of acts protected | 1.32 (Male),0.32(Female)  1.0 (Male), 2.12 (Female)  0.0  2  52  31.0 | GPS 2009  GPS 2009  GPS 2009  GPS 2009  GPS 2009  GPS 2009 | Size estimation based on reported multiple partners (>1 in the past 12 months) in the absence of transactional sex. Each of the other GPS estimates taken as the weighted average between men and women who reported multiple partnerships in the past 12 months. |
| **Partners of MP**  Population size estimation  HIV prevalence (%)  STI prevalence (%)  # of partners / year  # of sex acts / partner / year  % of acts protected | 0.20(Male),0.95(Female)  1.64  N/A  1  52  3.31 | GPS 2009  GPS 2009 (Married)  Assumption  GPS 2009  GPS 2009 | Reported consistent condom use and HIV prevalence taken as weighted average between men and women in this category (who are married or living with a partner). |
| **Low-risk heterosexual**  Population size estimation  HIV prevalence (%)  STI prevalence (%)  # of partners / year  # of sex acts / partner / year  % of acts protected | 42.3(Male) ,53.2(Female)  2.36  0.80  1  52  3.31 | Remainder of the population  GPS 2009  GPS 2009  GPS 2009  GPS 2009  GPS 2009 | Size estimation was in accordance with the GPS estimates.  HIV prevalence, STI prevalence, and reported consistent condom use taken as weighted average between men and women in this category. |
| **No risk**  Population size estimation  HIV prevalence (%)  STI prevalence (%)  # of partners / year  # of sex acts / partner / year  % of acts protected | Male(38.6),Female(30.4)  2.92  N/A  0  N/A  N/A | GPS 2009  GPS 2009 |  |
| **Medical injections**  Population size estimation  HIV prevalence (%)  STI prevalence (%)  # of partners / year  # of sex acts / partner / year  % of acts protected | Female(80.1),Male(70.5)  2.67  N/A  3 (injections/year)  1  95 | GPS 2007  GPS 2007 (overall prevalence)  GPS 2007  Set value  NFHS-3 (India-level data) |  |
| **Blood transfusions**  Population size estimation  HIV prevalence (%)  STI prevalence (%)  # of partners / year  # of sex acts / partner / year  % of acts protected | Female(0.11),Male(0.05)  2.67  N/A  1  1  100 | GPS 2009  GPS 2009 (overall prevalence)  NACO (NACP, 2004) | # of transfusions per 100 persons per year estimated from GPS 2009 assuming individuals are only transfused 1 in their life. |
| **% of adult males circumcised** | 17.5 | GPS 2009 |  |

ANC (Ante-natal clinic)[1,2,3,4,5]; MP (multiple partnerships); GPS (general population survey[1,6,7]); PBS (polling booth survey[1]); NACO (National AIDS Control Organization); FSW (female sex worker); HR-MSM (high-risk men who have sex with men); STI (sexually transmitted infection); NFHS-3 (National Family Health Survey Round 3[8]); BSS (Behavioural Surveillance Survey[9,10,11]); IBBA (Integrated Biological and Behavioural Assessment[1,12,13,14,15,16,17,18]). UNAIDS Country Progress Report 2010[4]

**Table S1c. Input values, sources, and assumptions for the Modes of Transmission Analysis for Belgaum district, India.**

| **Input descriptor** | **Default value (range)** | **Source(s)** | **Assumptions** |
| --- | --- | --- | --- |
| **IDU**  Population size (% of adult pop.)  HIV prevalence (%)  STI prevalence (%)  # of shared needles / year  # of shared needles/partner/ year  % of acts protected | 0(2.34;Male), 0(0.8;Female)  3.6  11.9  13  2  75 | GPS 2007 (PBS)  Sentinel surveillance state-level 2006  BSS 2006 (IDU)  BSS 2006 (IDU)  Assumption  BSS 2006 (IDU) | Size estimation based on % of men and women who report ever injecting illicit drugs (not just in the last 12 months).  All estimates from BSS 2007 (IDU) based on data from Bangalore. |
| **Partners of IDU**  Population size (% of adult female pop.)  HIV prevalence (%)  STI prevalence (%)  # of partners / year  # of sex acts / partner / year  % of acts protected | 0(0.6;Male), 0(1.42;Female)  1.3  N/A  1  52  18.3 | GPS 2007 (PBS)  GPS 2007 (Married)  Assumption  GPS 2007  GPS 2007 | Size estimation based on % of married men and women living with spouse, married living with other sexual partner, unmarried living with a sexual partner. Sexual partner in each case assumed to be of the opposite sex. |
| **FSW**  Population size (% of adult female pop.)  HIV prevalence (%)  STI prevalence (%)  # of partners / year  # of sex acts / partner / year  % of acts protected | 0.81  27.3  14.4  419  1  81.9 | Mapping data (2007)  IBBA 2008  IBBA 2008; Payana 2007-9  IBBA 2008;Payana 2007-9  Assumption  IBBA 2008;Payana 2007-9 | STI prevalence, # of partners/year, and consistent condom use based on the weighted average between rural FSWs (Payana) and urban FSWs (IBBA 2008).  Because the frequency of sex acts with repeat clients was not available, total client volume (with 1 exposure per client) was used.  % of acts protected additionally based on consistent condom use with repeat and occasional clients (weighted average) for urban and rural FSWs. |
| **Clients of FSWs**  Population size (% of adult male pop.)  HIV prevalence (%)  STI prevalence (%)  # of partners / year  # of sex acts / partner / year  % of acts protected | 0.3 (4.9,16.8)  6.2  8.3  19.2  59  46 | GPS 2007(PBS, indirect method)  IBBA 2007  IBBA 2007  IBBA 2007  Assumption  IBBA 2007 | # of partners/year includes only FSWs because this estimate is used to approximate whether total # of sex acts/client/year is close to that of FSWs.  # of sex acts/partner/year based on two findings; (1) # of sex acts for FSWs/year has to be near equal to # of sex acts for clients, and (2) median duration from time of last sex with a repeat FSW was 5 days in the cross-sectional IBBA 2007. |
| **Female partners of clients**  Population size (% of adult female pop.)  HIV prevalence (%)  STI prevalence (%)  # of partners / year  # of sex acts / partner / year  % of acts protected | 0.19(3.0,10.4)  0.90  N/A  1  120  6.0 | GPS (PBS,Indirect)  GPS 2007 (married Females)  Assumption  IBBA 2008  IBBA 2008 | Population size estimation based on % of men reporting currently married & living with spouse and unmarried men living with female partner (IBBA 2008) |
| **HR-MSM**  Population size (% of adult male pop.)  HIV prevalence (%)  STI prevalence (%)  # of partners / year  # of sex acts / partner / year  % of acts protected | 0.16  10.6  11.6  104  1  56.0 | Mapping data (2007)  IBBA  IBBA  IBBA  Assumption  IBBA |  |
| **Female partners of HR-MSM**  Population size (% of adult female pop.)  HIV prevalence (%)  STI prevalence (%)  # of partners / year  # of sex acts / partner / year  % of acts protected | 0.10  0.9  N/A  1  120  11 | IBBA  GPS (Married females)  Assumption  IBBA  IBBA | Population size estimation based on % of all MSM who are currently married & living with spouse and unmarried living with female partner |
| **Multiple partnerships (MP)**  Population size (% of adult male/female pop.)  HIV prevalence (%)  STI prevalence (%)  # of partners / year  # of sex acts / partner / year  % of acts protected | 1.25 (9.9; Male),0.12(1.5;Female)  4.77 (Male), 0.0 (Female)  56.0  2  12  23.0 | GPS 2007 (PBS)  GPS 2007  GPS 2007  GPS 2007  GPS 2007  GPS 2007 | Size estimation based on reported multiple partners (>1 in the past 12 months) in the absence of transactional sex. Each of the other GPS estimates taken as the weighted average between men and women who reported multiple partnerships in the past 12 months.  The PBS estimates for population size could include commercial sex work (unable to distinguish) so likely an overestimate of MP population size. |
| **Partners of MP**  Population size estimation  HIV prevalence (%)  STI prevalence (%)  # of partners / year  # of sex acts / partner / year  % of acts protected | 0.09(1.35;Male),0.77(6.1;Female)  1.43  N/A  1  52  16.9 | GPS 2007 (PBS)  GPS 2007 (Married)  Assumption  GPS 2007  GPS 2007 | Reported consistent condom use taken as weighted average between men and women in this category. |
| **Low-risk heterosexual**  Population size estimation  HIV prevalence (%)  STI prevalence (%)  # of partners / year  # of sex acts / partner / year  % of acts protected | 57.3(41.2,29.6; Male),  67.7(57.5,50.1;Female)  1.4  20.9  1  52  16.9 | Remainder of the pop. (GPS, PBS, indirect method)  GPS 2007  GPS 2007  GPS 2007  GPS 2007  GPS 2007 | Size estimation was in accordance with the GPS estimates.  HIV prevalence, STI prevalence, and reported consistent condom use taken as weighted average between men and women in this category.  Note that in the sensitivity analysis, the % of males and females changes based on different estimates of client size. |
| **No risk**  Population size estimation  HIV prevalence (%)  STI prevalence (%)  # of partners / year  # of sex acts / partner / year  % of acts protected | Male(40.9),Female(30.3)  2.3  N/A  0  N/A  N/A | GPS 2007  GPS 2007 |  |
| **Medical injections**  Population size estimation  HIV prevalence (%)  STI prevalence (%)  # of partners / year  # of sex acts / partner / year  % of acts protected | Male(72.5),Female(76.1)  1.43  N/A  4 (injections/year)  1  95 | GPS 2007  GPS 2007 (overall prevalence)  GPS 2007  Set value  NFHS-3 (India-level data) |  |
| **Blood transfusions**  Population size estimation  HIV prevalence (%)  STI prevalence (%)  # of partners / year  # of sex acts / partner / year  % of acts protected | Male(0.03),Female(0.10)  1.43  N/A  1  1  100 | GPS 2007  GPS 2007 (overall prevalence)  NACO (NACP, 2004) | # of transfusions per 100 persons per year estimated from GPS 2007 assuming individuals are only transfused 1 in their life. |
| **% of adult males circumcised** | 10.0 | GPS 2007 |  |

ANC (Ante-natal clinic)[1,2,3,4,5]; MP (multiple partnerships); GPS (general population survey[1,6,7]); PBS (polling booth survey[1]); NACO (National AIDS Control Organization); FSW (female sex worker); HR-MSM (high-risk men who have sex with men); STI (sexually transmitted infection); NFHS-3 (National Family Health Survey Round 3[8]); BSS (Behavioural Surveillance Survey[9,10,11]); IBBA (Integrated Biological and Behavioural Assessment[1,12,13,14,15,16,17,18]). UNAIDS Country Progress Report 2010[4]

**Table S1d**. Input values, sources, and assumptions for the Modes of Transmission Analysis for select district Ganjam, India.

| **Input descriptor** | **Default value (range)** | **Source(s)** | **Notes** |
| --- | --- | --- | --- |
| **IDU**  Population size (% of adult pop.) | 0.0 | NGO mapping |  |
| **Partners of IDU**  Population size (% of adult female pop.) | 0.0 |  |  |
| **FSW**  Population size (% of adult female pop.)  HIV prevalence (%)  STI prevalence (%)  # of partners / year  # of sex acts / partner / year  % of acts protected | 0.033  1.0  33.5  452  1  58.7 | NGO Mapping data  NACO state-level estimates 2006  BSS 2006 (FSW) state-level  BSS 2006 (FSW) state-level  No data source  BSS 2006 (FSW) state-level | Assumed prevalence of GUD was 50% of all cases of symptomatic STIs in last 1 year  % of acts protected based on consistent condom use with clients in the last 3 months. |
| **Clients of FSWs**  Population size (% of adult male pop.)  HIV prevalence (%)  STI prevalence (%)  # of partners / year  # of sex acts / partner / year  % of acts protected | 1.9  0.96  7.8  15.6  1  62.8 | BSS 2006 (General pop.) state-level  NFHS-3 national-level  BSS 2006 (Clients) state-level  BSS 2006 (Clients) state-level  Assumption, no data  BSS 2006 (Clients) state-level | Client population based on % of men reporting having paid for sex in last 1 year (BSS 2006)  # of sex acts/partner/year approximated to almost equal total # of FSW sex acts / year  % of acts protected based on consistent condom use with FSWs in the last 3 months. |
| **Female partners of clients**  Population size (% of adult female pop.)  HIV prevalence (%)  STI prevalence (%)  # of partners / year  # of sex acts / partner / year  % of acts protected | 1.11  3.25  N/A  1  52  18.4 | BSS 2006 (Clients) state-level  ANC surveillance 2006  Assumption  Belgaum GPS  BSS 2006 (General Population) | Population size estimation based on % of men reporting currently married & living with spouse and unmarried men living with female partner.  Number of sex acts in a married or cohabitating relationship estimated from median # frequency of sex reported by men and women in the Belgaum GPS |
| **HR-MSM**  Population size (% of adult male pop.) | 0.0 | NGO mapping |  |
| **Female partners of HR-MSM**  Population size (% of adult female pop.) | 0.0 |  |  |
| **Multiple partnerships (MP)**  Population size (% of adult male/female pop.)  HIV prevalence (%)  STI prevalence (%)  # of partners / year  # of sex acts / partner / year  % of acts protected | Male (6.7),Female (4.3)  3.25  5.9  2  26  19.7 | BSS 2006 (General pop.) state-level  ANC surveillance 2006  BSS 2006 (General pop.) state-level  NFHS-3 national-level  Assumption, no data  BSS 2006 (General pop.) state-level | Size estimation from the BSS based on reporting sex with non-regular partners. May include individuals with 1 partner in the last year if he/she were a casual sex partner (i.e. not a regular partner). |
| **Partners of MP**  Population size estimation  HIV prevalence (%)  STI prevalence (%)  # of partners / year  # of sex acts / partner / year  % of acts protected | Males (3.2),Females(4.2)  3.25  N/A  1  52  18.4 | NFHS-3 national-level  Assumption (ANC prevalence 2006)  Assumption  Belgaum GPS  BSS 2006 (General pop.) state-level | National-level estimates of marriage among adult men and women used to estimate population size.  Consistent condom use among spouse/regular partner in the last 12 months. |
| **Low-risk heterosexual**  Population size estimation  HIV prevalence (%)  STI prevalence (%)  # of partners / year  # of sex acts / partner / year  % of acts protected | Male (80.7),Female(80.4)  3.25  0  1  52  18.4 | Remainder of population  Assumption (overall prevalence)  Assumption, no data  Assumption  Belgaum GPS  BSS 2006 (General pop.) state-level | Consistent condom use among spouse/regular partner in the last 12 months. |
| **No risk**  Population size estimation  HIV prevalence (%)  STI prevalence (%)  # of partners / year  # of sex acts / partner / year  % of acts protected | Male(7.5),Female(10.0)  3.25  N/A  0  N/A  N/A | NFHS-3 National data  Assumption (overall prevalence) |  |
| **Medical injections**  Population size estimation  HIV prevalence (%)  STI prevalence (%)  # of partners / year  # of sex acts / partner / year  % of acts protected | Male(35.7),Female(38.5)  3.25  N/A  2 (injections/year)  1  95 | NFHS-3 National data  Assumption (overall prevalence)  NFHS-3 National data  Set value  NFHS-3 National data |  |
| **Blood transfusions**  Population size estimation  HIV prevalence (%)  STI prevalence (%)  # of partners / year  # of sex acts / partner / year  % of acts protected | Male(0.1),Female(0.13)  3.25  N/A  1  1  100 | NFHS-3 National data  Assumption (overall prevalence)  Set value  Set value  NACO (NACP, 2004) | # of transfusions per 100 persons per year estimated from NFHS-3 assuming individuals are only transfused 1 in their life. |
| **% of adult males circumcised** | 12.7 | NFHS-3 National data |  |

ANC (Ante-natal clinic)[1,2,3,4,5]; MP (multiple partnerships); GPS (general population survey[1,6,7]); PBS (polling booth survey[1]); NACO (National AIDS Control Organization); FSW (female sex worker); HR-MSM (high-risk men who have sex with men); STI (sexually transmitted infection); NFHS-3 (National Family Health Survey Round 3[8]); BSS (Behavioural Surveillance Survey[9,10,11]); IBBA (Integrated Biological and Behavioural Assessment[1,12,13,14,15,16,17,18]). UNAIDS Country Progress Report 2010[4]

**Table S1e. Input values, sources, and assumptions for the Modes of Transmission Analysis for district Shimoga, India**.

| **Input descriptor** | **Default value (range)** | **Source(s)** | **Notes** |
| --- | --- | --- | --- |
| **IDU**  Population size (% of adult pop.) | 0.0 | NGO Mapping data |  |
| **Partners of IDU**  Population size (% of adult female pop.) | 0.0 |  |  |
| **FSW**  Population size (% of adult female pop.)  HIV prevalence (%)  STI prevalence (%)  # of partners / year  # of sex acts / partner / year  % of acts protected | 0.32  9.0  7.6  520  1  73.0 | Mapping data 2007  IBBA 2008  IBBA 2008  IBBA 2008  Assumption  IBBA 2008 | Because the frequency of sex acts with repeat clients was not available, total client volume (with 1 exposure per client) was used.  % of acts protected based on consistent condom use with repeat and occasional clients (weighted average). |
| **Clients of FSWs**  Population size (% of adult male pop.)  HIV prevalence (%)  STI prevalence (%)  # of partners / year  # of sex acts / partner / year  % of acts protected | 10.2  2.4  4.2  15  1  73.0 | indirect method  IBBA 2008  IBBA 2008  IBBA 2008  Assumption  IBBA 2008 | # of sex acts/partner/year approximated to almost equal total # of FSW sex acts / year |
| **Female partners of clients**  Population size (% of adult female pop.)  HIV prevalence (%)  STI prevalence (%)  # of partners / year  # of sex acts / partner / year  % of acts protected | 6.4  1.0  N/A  1  120  5.0 | Assumption (ANC prev.)  Assumption  IBBA 2008  IBBA 2008 | Population size estimation based on % of men reporting currently married & living with spouse and unmarried men living with female partner (IBBA 2008) |
| **HR-MSM**  Population size (% of adult male pop.)  HIV prevalence (%)  STI prevalence (%)  # of partners / year  # of sex acts / partner / year  % of acts protected | 0.07  9.9  3.2  104  1  52.0 | Mapping 2007  IBBA  IBBA  IBBA  Assumption  IBBA |  |
| **Female partners of HR-MSM**  Population size (% of adult female pop.)  HIV prevalence (%)  STI prevalence (%)  # of partners / year  # of sex acts / partner / year  % of acts protected | 0.03  1.0  N/A  1  120  9.0 | Assumption (ANC prev.)  Assumption  IBBA  IBBA | Population size estimation based on % of all MSM who are currently married & living with spouse and unmarried living with female partner |
| **Multiple partnerships (MP)**  Population size (% of adult male/female pop.)  HIV prevalence (%)  STI prevalence (%)  # of partners / year  # of sex acts / partner / year  % of acts protected | Male (3.1),Female (0.8)  1.0  2.5  2  12  55.0 | BSS 2006 (State-level est.)  Assumption (ANC prev.)  BSS 2006 (Total pop.; state-level est.)  Assumption  Assumption  BSS 2006 (state-level est.) |  |
| **Partners of MP**  Population size estimation  HIV prevalence (%)  STI prevalence (%)  # of partners / year  # of sex acts / partner / year  % of acts protected | Males (0.6),Females(1.9)  1.0  N/A  1  52 | NFHS-3  Assumption (ANC prevalence)  Assumption  Belgaum GPS  BSS 2006 (General Population) | Number of sex acts in a married or cohabitating relationship estimated from median # frequency of sex reported by men and women in the Belgaum GPS |
| **Low-risk heterosexual**  Population size estimation  HIV prevalence (%)  STI prevalence (%)  # of partners / year  # of sex acts / partner / year  % of acts protected | Male (87.8),Female(86.6)  1.0  0  1  52  11.0 | Remainder of the pop.  Assumption (ANC prev.)  Assumption, no data  Assumption  Belgaum GPS  BSS 2006 (state-level est.) |  |
| **No risk**  Population size estimation  HIV prevalence (%)  STI prevalence (%)  # of partners / year  # of sex acts / partner / year  % of acts protected | Male(7.5),Female(10.0)  1.0  N/A  0  N/A  N/A | National-level est.  Assumption (ANC prev.) |  |
| **Medical injections**  Population size estimation  HIV prevalence (%)  STI prevalence (%)  # of partners / year  # of sex acts / partner / year  % of acts protected | Male(53.5),Female(53.3)  1.0  N/A  2 (injections/year)  1  95 | NFHS-3/National-level est.  Assumption (ANC prev.)  NFHS-3/National-level est.  Set value  NFHS-3/National level est. |  |
| **Blood transfusions**  Population size estimation  HIV prevalence (%)  STI prevalence (%)  # of partners / year  # of sex acts / partner / year  % of acts protected | Male(0.07),Female(0.14)  1.0  N/A  1  1  100 | NFHS-3/National-level est.  Assumption (ANC prev.)  NACO (NACP, 2004) | # of transfusions per 100 persons per year estimated from NFHS-3 assuming individuals are only transfused 1 in their life. |
| **% of adult males circumcised** | 12.7 | National level estimate |  |

ANC (Ante-natal clinic)[1,2,3,4,5]; MP (multiple partnerships); GPS (general population survey[1,6,7]); PBS (polling booth survey[1]); NACO (National AIDS Control Organization); FSW (female sex worker); HR-MSM (high-risk men who have sex with men); STI (sexually transmitted infection); NFHS-3 (National Family Health Survey Round 3[8]); BSS (Behavioural Surveillance Survey[9,10,11]); IBBA (Integrated Biological and Behavioural Assessment[1,12,13,14,15,16,17,18]). UNAIDS Country Progress Report 2010[4]

**Table S1f. Input values, sources, and assumptions for the Modes of Transmission Analysis for district Sikar, India**.

| **Input descriptor** | **Default value (range)** | **Source(s)** | **Notes** |
| --- | --- | --- | --- |
| **IDU**  Population size (% of adult pop.)  HIV prevalence (%)  STI prevalence (%)  # of partners / year  # of sex acts / partner / year  % of acts protected | 0.01  4.6  11.9  90  2  50 | NGO mapping  NACO estimates 2006 state data  BSS 2006 (IDU)  BSS 2006 (IDU)  Assumption  BSS 2006 (IDU) | No district level data available.  BSS for IDU population restricted to 10 cities across India.  # of partners/year based on median frequency of sharing /using needle previously used by someone else (occasionally per month – assumed to be 7⋅5 injections / month)  % of acts protected based on median frequency of cleaning used needle prior to use (half the time per month) |
| **Partners of IDU**  Population size (% of adult female pop.)  HIV prevalence (%)  STI prevalence (%)  # of partners / year  # of sex acts / partner / year  % of acts protected | 0.005  0.98  N/A  1  52  21.0 | BSS 2006 (IDU) state data  GPS 2004  Belgaum GPS  BSS 2006 (General pop.) state data | No district level data available.  Population size based on % of married men living with spouse, married men living with other sexual partner, unmarried men living with a sexual partner. Sexual partner in each case assumed to be female.  Number of sex acts in a married or cohabitating relationship estimated from median # frequency of sex reported by men and women in the Belgaum GPS  Consistent condom use reported by females with spouse/regular partner in the last 12 months. |
| **FSW**  Population size (% of adult female pop.)  HIV prevalence (%)  STI prevalence (%)  # of partners / year  # of sex acts / partner / year  % of acts protected | 0.05  0.98  20.2  411  1  85.2 | NGO Mapping data  GPS 2004  BSS 2006 (FSW) state-level  BSS 2006 (FSW) state-level  No data source  BSS 2006 (FSW) state-level | Assumed prevalence of GUD was 50% of all cases of symptomatic STIs in last 1 year  % of acts protected based on consistent condom use with clients in the last 3 months. |
| **Clients of FSWs**  Population size (% of adult male pop.)  HIV prevalence (%)  STI prevalence (%)  # of partners / year  # of sex acts / partner / year  % of acts protected | 1.6  0.96  28.1  14.8  15  84.2 | BSS 2006 (General pop.) state-level  NFHS-3 national-level  BSS 2006 (Clients) state-level  BSS 2006 (Clients) state-level  Assumption, no data  BSS 2006 (Clients) state-level | Client population based on % of men reporting having paid for sex in last 1 year (BSS 2006)  # of sex acts/partner/year approximated to almost equal total # of FSW sex acts / year  % of acts protected based on consistent condom use with FSWs in the last 3 months. |
| **Female partners of clients**  Population size (% of adult female pop.)  HIV prevalence (%)  STI prevalence (%)  # of partners / year  # of sex acts / partner / year  % of acts protected | 0.93  0.98  N/A  1  52  21 | BSS 2006 (Clients) state level  GPS 2004  Assumption  Belgaum GPS  BSS 2006 (General Pop.) state level | Population size estimation based on % of men reporting currently married & living with spouse and unmarried men living with female partner.  Number of sex acts in a married or cohabitating relationship estimated from median # frequency of sex reported by men and women in the Belgaum GPS |
| **HR-MSM**  Population size (% of adult male pop.)  HIV prevalence (%)  STI prevalence (%)  # of partners / year  # of sex acts / partner / year  % of acts protected | 0.03  0.0  3.2  78  1  71.4 | NGO mapping  NACO 2006 state level  BSS 2006 (MSM) national level  IBBA 2006-2007 national level  Assumption, no data  BSS 2006 (MSM) national level | IBBA in 4 states (Karnataka, Andhra Pradesh, Maharastra, Tamil Nadu)  BSS among MSM restricted to 10 cities.  % of sex acts protected based on consistent condom use among HR-MSM in the last 6 months. |
| **Female partners of HR-MSM**  Population size (% of adult female pop.)  HIV prevalence (%)  STI prevalence (%)  # of partners / year  # of sex acts / partner / year  % of acts protected | 0.02  0.98  N/A  1  26  21.0 | BSS 2006 (MSM) national level  GPS 2004  Assumption  Assumption  BSS 2006 (Gen pop.) state level | Population size estimation based on % of all MSM who are currently married & living with spouse and unmarried living with female partner.  Assumed # of sex acts with female sex partner approximately half that of non high risk MSM-female relationships. |
| **Multiple partnerships (MP)**  Population size (% of adult male/female pop.)  HIV prevalence (%)  STI prevalence (%)  # of partners / year  # of sex acts / partner / year  % of acts protected | Male (8.0),Female (1.5)  0.98  2.2  2  26  40.7 | BSS 2006 (General pop.) state-level  GPS 2004  BSS 2006 (General pop.) state-level  NFHS-3 national-level  Assumption, no data  BSS 2006 (General pop.) state-level | Size estimation from the BSS based on reporting sex with non-regular partners. May include individuals with 1 partner in the last year if he/she were a casual sex partner (i.e. not a regular partner). |
| **Partners of MP**  Population size estimation  HIV prevalence (%)  STI prevalence (%)  # of partners / year  # of sex acts / partner / year  % of acts protected | Males (1.1),Females(4.9)  0.98  N/A  1  52  21.0 | NFHS-3 national-level  GPS 2004  Assumption  Belgaum GPS  BSS 2006 (General pop.) state-level | National-level estimates of marriage among adult men and women are used to estimate population size.  Consistent condom use among spouse/regular partner in the last 12 months. |
| **Low-risk heterosexual**  Population size estimation  HIV prevalence (%)  STI prevalence (%)  # of partners / year  # of sex acts / partner / year  % of acts protected | Male (81.8),Female(82.6)  0.98  0  1  52  21.0 | Remainder of population  GPS 2004  Assumption, no data  Assumption  Belgaum GPS  BSS 2006 (General pop.) state-level | Consistent condom use among spouse/regular partner in the last 12 months. |
| **No risk**  Population size estimation  HIV prevalence (%)  STI prevalence (%)  # of partners / year  # of sex acts / partner / year  % of acts protected | Male(7.5),Female(10.0)  0.98  N/A  0  N/A  N/A | NFHS-3 National data  Assumption (overall prevalence) |  |
| **Medical injections**  Population size estimation  HIV prevalence (%)  STI prevalence (%)  # of partners / year  # of sex acts / partner / year  % of acts protected | Male(35.7),Female(38.5)  0.98  N/A  2 (injections/year)  1  95 | NFHS-3 National data  Assumption (overall prevalence)  NFHS-3 National data  Set value  NFHS-3 National data |  |
| **Blood transfusions**  Population size estimation  HIV prevalence (%)  STI prevalence (%)  # of partners / year  # of sex acts / partner / year  % of acts protected | Male(0.1),Female(0.13)  0.98  N/A  1  1  100 | NFHS-3 National data  Assumption (overall prevalence)  Set value  Set value  NACO (NACP, 2004) | # of transfusions per 100 persons per year estimated from NFHS-3 assuming individuals are only transfused 1 in their life. |
| **% of adult males circumcised** | 12.7 | NFHS-3 National data |  |

ANC (Ante-natal clinic)[1,2,3,4,5]; MP (multiple partnerships); GPS (general population survey[1,6,7]); PBS (polling booth survey[1]); NACO (National AIDS Control Organization); FSW (female sex worker); HR-MSM (high-risk men who have sex with men); STI (sexually transmitted infection); NFHS-3 (National Family Health Survey Round 3[8]); BSS (Behavioural Surveillance Survey[9,10,11]); IBBA (Integrated Biological and Behavioural Assessment[1,12,13,14,15,16,17,18]). UNAIDS Country Progress Report 2010[4]

**Table S1g. Input values, sources, and assumptions for the Modes of Transmission Analysis for district Varanasi, India**.

| **Input descriptor** | **Default value (range)** | **Source(s)** | **Notes** |
| --- | --- | --- | --- |
| **IDU**  Population size (% of adult pop.)  HIV prevalence (%)  STI prevalence (%)  # of partners / year  # of sex acts / partner / year  % of acts protected | 0.04  3.2  11.9  90  2  50 | NGO mapping  NACO estimates 2006 state data  BSS 2006 (IDU)  BSS 2006 (IDU)  Assumption  BSS 2006 (IDU) | No district level data available.  BSS for IDU population restricted to 10 cities across India.  # of partners/year based on median frequency of sharing /using needle previously used by someone else (occasionally per month – assumed to be 7.5 hits / month)  % of acts protected based on median frequency of cleaning used needle prior to use (half the time per month) |
| **Partners of IDU**  Population size (% of adult female pop.)  HIV prevalence (%)  STI prevalence (%)  # of partners / year  # of sex acts / partner / year  % of acts protected | 0.02  0.25  N/A  1  52  26.1 | BSS 2006 (IDU) state data  GPS 2004  Belgaum GPS  BSS 2006 (General pop.) state data | No district level data available.  Population size based on % of married men living with spouse, married men living with other sexual partner, unmarried men living with a sexual partner. Sexual partner in each case assumed to be female.  Number of sex acts in a married or cohabitating relationship estimated from median # frequency of sex reported by men and women in the Belgaum GPS  Consistent condom use reported by females with spouse/regular partner in the last 12 months. |
| **FSW**  Population size (% of adult female pop.)  HIV prevalence (%)  STI prevalence (%)  # of partners / year  # of sex acts / partner / year  % of acts protected | 0.08  0.8  23.0  494  1  72.6 | NGO Mapping data  FSW clinic surveillance 2006  BSS 2006 (FSW) state-level  BSS 2006 (FSW) state-level  No data source  BSS 2006 (FSW) state-level | Assumed prevalence of GUD was 50% of all cases of symptomatic STIs in last 1 year  % of acts protected based on consistent condom use with clients in the last 3 months. |
| **Clients of FSWs**  Population size (% of adult male pop.)  HIV prevalence (%)  STI prevalence (%)  # of partners / year  # of sex acts / partner / year  % of acts protected | 1.8  0.96  4.0  10  1  71.2 | BSS 2006 (General pop.) state-level  NFHS-3 national-level  BSS 2006 (Clients) state-level  BSS 2006 (Clients) state-level  Assumption, no data  BSS 2006 (Clients) state-level | Client population based on % of men reporting having paid for sex in last 1 year (BSS 2006)  # of sex acts/partner/year approximated to almost equal total # of FSW sex acts / year  % of acts protected based on consistent condom use with FSWs in the last 3 months. |
| **Female partners of clients**  Population size (% of adult female pop.)  HIV prevalence (%)  STI prevalence (%)  # of partners / year  # of sex acts / partner / year  % of acts protected | 1.0  0.25  N/A  1  52  26.1 | BSS 2006 (Clients) state-level  PPTCT surveillance 2008  Assumption  Belgaum GPS  BSS 2006 (General Population) | Population size estimation based on % of men reporting currently married & living with spouse and unmarried men living with female partner.  Number of sex acts in a married or cohabitating relationship estimated from median # frequency of sex reported by men and women in the Belgaum GPS |
| **HR-MSM**  Population size (% of adult male pop.)  HIV prevalence (%)  STI prevalence (%)  # of partners / year  # of sex acts / partner / year  % of acts protected | 0.07  2.0  3.2  78  1  71.4 | NGO mapping  NACO 2006 state level  BSS 2006 (MSM) national level  IBBA 2006-2007 national level  Assumption, no data  BSS 2006 (MSM) national level | IBBA in 4 states (Karnataka, Andhra Pradesh, Maharastra, Tamil Nadu)  BSS among MSM restricted to 10 cities.  % of sex acts protected based on consistent condom use among HR-MSM in the last 6 months. |
| **Female partners of HR-MSM**  Population size (% of adult female pop.)  HIV prevalence (%)  STI prevalence (%)  # of partners / year  # of sex acts / partner / year  % of acts protected | 0.04  0.25  N/A  1  26  21.0 | BSS 2006 (MSM) national level  PPTCT prevalence 2008  Assumption  Assumption  BSS 2006 (Gen pop.) state level | Population size estimation based on % of all MSM who are currently married & living with spouse and unmarried living with female partner.  Assumed # of sex acts with female sex partner approximately half that of non high risk MSM-female relationships. |
| **Multiple partnerships (MP)**  Population size (% of adult male/female pop.)  HIV prevalence (%)  STI prevalence (%)  # of partners / year  # of sex acts / partner / year  % of acts protected | Male (9.2),Female (0.4)  0.25  1.1  2  26  28.8 | BSS 2006 (General pop.) state-level  PPTCT surveillance 2008  BSS 2006 (General pop.) state-level  NFHS-3 national-level  Assumption, no data  BSS 2006 (General pop.) state-level | Size estimation from the BSS based on reporting sex with non-regular partners. May include individuals with 1 partner in the last year if he/she were a casual sex partner (i.e. not a regular partner). |
| **Partners of MP**  Population size estimation  HIV prevalence (%)  STI prevalence (%)  # of partners / year  # of sex acts / partner / year  % of acts protected | Males (0.3),Females(5.7)  0.25  N/A  1  52  19.5 | NFHS-3 national-level  PPTCT prevalence 2008  Assumption  Belgaum GPS  BSS 2006 (General pop.) state-level | National-level estimates of marriage among adult men and women used to estimate population size.  Consistent condom use among spouse/regular partner in the last 12 months. |
| **Low-risk heterosexual**  Population size estimation  HIV prevalence (%)  STI prevalence (%)  # of partners / year  # of sex acts / partner / year  % of acts protected | Male (81.1),Female(82.8)  0.25  1.1  1  52  19.5 | Remainder of population  ANC prevalence 2006  BSS (General pop.) state level  Assumption  Belgaum GPS  BSS 2006 (General pop.) state-level | Consistent condom use among spouse/regular partner in the last 12 months. |
| **No risk**  Population size estimation  HIV prevalence (%)  STI prevalence (%)  # of partners / year  # of sex acts / partner / year  % of acts protected | Male(7.5),Female(10.0)  0.25  N/A  0  N/A  N/A | NFHS-3 National data  ANC prevalence 2006 |  |
| **Medical injections**  Population size estimation  HIV prevalence (%)  STI prevalence (%)  # of partners / year  # of sex acts / partner / year  % of acts protected | Male(35.7),Female(38.5)  0.25  N/A  2 (injections/year)  1  95 | NFHS-3 National data  Assumption (overall prevalence)  NFHS-3 National data  Set value  NFHS-3 National data |  |
| **Blood transfusions**  Population size estimation  HIV prevalence (%)  STI prevalence (%)  # of partners / year  # of sex acts / partner / year  % of acts protected | Male(0.1),Female(0.13)  0.25  N/A  1  1  100 | NFHS-3 National data  Assumption (overall prevalence)  Set value  Set value  NACO (NACP, 2004) | # of transfusions per 100 persons per year estimated from NFHS-3 assuming individuals are only transfused 1 in their life. |
| **% of adult males circumcised** | 12.7 | NFHS-3 National data |  |

ANC (Ante-natal clinic)[1,2,3,4,5]; MP (multiple partnerships); GPS (general population survey[1,6,7]); PBS (polling booth survey[1]); NACO (National AIDS Control Organization); FSW (female sex worker); HR-MSM (high-risk men who have sex with men); STI (sexually transmitted infection); NFHS-3 (National Family Health Survey Round 3[8]); BSS (Behavioural Surveillance Survey[9,10,11]); IBBA (Integrated Biological and Behavioural Assessment[1,12,13,14,15,16,17,18]). UNAIDS Country Progress Report 2010[4]

**Table S1h. Input values, sources, and assumptions for the Modes of Transmission Analysis for district Belgaum, India: state level estimates** for sensitivity analysis.

| **Input descriptor** | **Default value (range)** | **Source(s)** | **Assumptions** |
| --- | --- | --- | --- |
| **IDU**  Population size (% of adult pop.)  HIV prevalence (%)  STI prevalence (%)  # of shared needles / year  # of shared needles/partner/ year  % of acts protected | 0.06  9.2  11.9  90  2  50 |  | Based on national level estimates. |
| **Partners of IDU**  Population size (% of adult female pop.)  HIV prevalence (%)  STI prevalence (%)  # of partners / year  # of sex acts / partner / year  % of acts protected | 0.04  0.54  N/A  1  52  13.8 | BSS 2006  NFHS-3  Assumption  Assumption  BSS 2006 | Size estimation based on % of married men and women living with spouse, married living with other sexual partner, unmarried living with a sexual partner. Sexual partner in each case assumed to be of the opposite sex. |
| **FSW**  Population size (% of adult female pop.)  HIV prevalence (%)  STI prevalence (%)  # of partners / year  # of sex acts / partner / year  % of acts protected | 0.79  5.3  33.2  328  1  87.4 | Mapping data  Sentinel Surveillance 2008  BSS 2006  BSS 2006  Assumption  BSS 2006 | % STI prevalence based on 50% of reported STI symptoms. |
| **Clients of FSWs**  Population size (% of adult male pop.)  HIV prevalence (%)  STI prevalence (%)  # of partners / year  # of sex acts / partner / year  % of acts protected | 3.0  0.96  16.6  14.4  6  87.4 | BSS 2006  NFHS-3  BSS 2006  BSS 2006  Assumption  BSS 2006 |  |
| **Female partners of clients**  Population size (% of adult female pop.)  HIV prevalence (%)  STI prevalence (%)  # of partners / year  # of sex acts / partner / year  % of acts protected | 1.74  0.54  N/A  1  52  13.8 | BSS 2006  NFHS-3  Assumption  Assumption  Assumption  BSS 2006 | Population size estimation based on % of men reporting currently married & living with spouse and unmarried men living with female partner |
| **HR-MSM**  Population size (% of adult male pop.)  HIV prevalence (%)  STI prevalence (%)  # of partners / year  # of sex acts / partner / year  % of acts protected | 0.16  13.0  3.2  78  1  71.4 | Mapping data (2007)  Sentinel Surveillance | National estimates used for all parameters except population size and HIV prevalence. |
| **Female partners of HR-MSM**  Population size (% of adult female pop.)  HIV prevalence (%)  STI prevalence (%)  # of partners / year  # of sex acts / partner / year  % of acts protected | 0.09  0.54  N/A  1  26  13.8 | BSS 2006  NFHS-3  Assumption  Assumption  BSS 2006 | Population size estimation based on % of all adult men who are currently married & living with spouse and unmarried living with female partner. |
| **Multiple partnerships (MP)**  Population size (% of adult male/female pop.)  HIV prevalence (%)  STI prevalence (%)  # of partners / year  # of sex acts / partner / year  % of acts protected | Male (3.1),Female (0.8)  0.69  2.5  2  12  54.5 | BSS 2006  NFHS-3/Assumption  BSS 2006 (total pop.)  Assumption  Assumption  BSS 2006 | Size estimation based on report of non-regular partner in the BSS 2006 General Population, but includes sex work. |
| **Partners of MP**  Population size estimation  HIV prevalence (%)  STI prevalence (%)  # of partners / year  # of sex acts / partner / year  % of acts protected | Males (0.6),Females(1.8)  0.69  N/A  1  52  13.8 | BSS 2006  NFHS-3/Assumption  Assumption  Assumption  BSS 2006 | Size estimation based on reported currently married or cohabitating with a partner. Partner assumed to be of opposite sex. |
| **Low-risk heterosexual**  Population size estimation  HIV prevalence (%)  STI prevalence (%)  # of partners / year  # of sex acts / partner / year  % of acts protected | Male (85.6),Female (84.7)  0.69  2.5  1  52  13.8 | Remainder of the population  NFHS-3/Assumption  BSS 2006 (Total pop.) |  |
| **No risk**  Population size estimation  HIV prevalence (%)  STI prevalence (%)  # of partners / year  # of sex acts / partner / year  % of acts protected | Male(7.5),Female(10.0)  0.69  N/A  0  N/A  N/A | NFHS-3 National data  NFHS-3 (state overall prevalence) |  |
| **Medical injections**  Population size estimation  HIV prevalence (%)  STI prevalence (%)  # of partners / year  # of sex acts / partner / year  % of acts protected | Male(35.7),Female(38.5)  0.69  N/A  2 (injections/year)  1  95 | NFHS-3 National-data  NFHS-3 (state overall prevalence)  NFHS-3 National-data  NFHS-3 National data  Set value  NFHS-3 National data | Utilized national-level estimates. |
| **Blood transfusions**  Population size estimation  HIV prevalence (%)  STI prevalence (%)  # of partners / year  # of sex acts / partner / year  % of acts protected | Male(0.1),Female(0.13)  0.69  N/A  1  1  100 | NFHS-3 National data  NFHS-3 (state overall prevalence)  Set value  Set value  NACO (NACP, 2004) | Utilized national-level estimates. |
| **% of adult males circumcised** | 12.7 | NFHS-3 National data |  |

ANC (Ante-natal clinic)[1,2,3,4,5]; MP (multiple partnerships); GPS (general population survey[1,6,7]); PBS (polling booth survey[1]); NACO (National AIDS Control Organization); FSW (female sex worker); HR-MSM (high-risk men who have sex with men); STI (sexually transmitted infection); NFHS-3 (National Family Health Survey Round 3[8]); BSS (Behavioural Surveillance Survey[9,10,11]); IBBA (Integrated Biological and Behavioural Assessment[1,12,13,14,15,16,17,18]). UNAIDS Country Progress Report 2010[4]

References

1. India Health Action Trust (2010) HIV/AIDS situation and response in Karnataka: Epidemiological appraisal using data triangulation. Bangalore.

2. India Health Action Trust (2010) HIV/AIDS situation and response in Uttar Pradesh: Epidemiological appraisal using data triangulation. Bangalore.

3. National AIDS Control Organization (2009) Annual report 2008-2009. New Delhi: Ministry of Health

& Family Welfare, Government of India. Available:

http://www.nacoonline.org/Quick_Links/Publication/ME_and_Research_Surveillance/ .

Accessed 2 October 2011.

4. National AIDS Control Organization (2010) UNAIDS Country Progress Report: India. Available:

<http://www.unaids.org/en/dataanalysis/monitoringcountryprogress/2010progressreportssubmittedbycountr>

ies/ . Accessed 2 October 2011.

5. National AIDS Control organization. Press release: HIV declining in India. Delhi: Ministry of health

and family welfare, Government of India, 2010.

http://www.nacoonline.org/Quick_Links/Publication/ME_and_Research_Surveillance/ .

Accessed 2 October 2011.

6. Rajaram S, Bradley J, Alary M, Ramesh B, Washington R, et al. (2010) HIV and STIs in Bagalkot

district, Karnataka, India. A general population survey. Bangalore. Available:

http://www.khpt.org/charme.html. Accessed 2 October 2011.

7. Rajaram S, Sangameshwar S, Jayachandran A, Bradley J, Alary M, et al. (2008) HIV and STIs in

Belgaum district, Karnataka, India. A general population survey. Bangalore. Available:

<http://www.khpt.org/charme.html>. Accessed 2 October 2011.

8. National Family Health Survey India (2006) NFHS-3 country and state reports. Delhi. http://www.nfhsindia.org/nfhs3_national_report.html (accessed Jun 20, 2011)

9. Ministry of health and family welfare National behavioural surveillance survey 2006: female sex

workers and their clients. Delhi: National AIDS Control Organization, Government of India. Available:

<http://www.nacoonline.org/Quick_Links/Publication/ME_and_Research_Surveillance/>. Accessed 2

October 2011.

10. Ministry of health and family welfare National behavioural surveillance survey 2006: men who have

sex with men and injecting drug users. Delhi: National AIDS Control Organization, Government of India.

Available: http://www.nacoonline.org/Quick_Links/Publication/ME_and_Research_Surveillance/ .

Accessed 2 October 2011.

11. Ministry of health and family welfare National behavioural surveillance survey 2006: general

population. Delhi: National AIDS Control Organization, Government of India. Available:

http://www.nacoonline.org/Quick_Links/Publication/ME_and_Research_Surveillance/ . Accessed 2

October 2011.

12. Ramakrishnan L, Gautam A, Goswami P, Kallam S, Adhikary R, et al. (2010) Programme coverage, condom use and STI treatment among FSWs in a large-scale HIV prevention programme: results from cross-sectional surveys in 22 districts in southern India. Sex Transm Infect 86: I62-I68.

13. Brahmam GNV, Kodavallaa V, Rajkumar H, Rachakulla HK, Kallam S, et al. (2008) Sexual

practices, HIV and sexually transmitted infections among self-identified men who have sex with men in four high HIV prevalence states of India. AIDS 22: S45-S57.

14. Ramesh BM, Beattie TSH, Shajy I, Washington R, Jagannathan L, et al. (2010) Changes in risk

behaviours and prevalence of sexually transmitted infections following HIV preventive interventions among female sex workers in five districts in Karnataka state, south India. Sex Transm Infect 86: I17-I24.

15. Saidel T, Adhikary R, Mainkar M, Dale J, Loo V, et al. (2008) Baseline integrated behavioural and

biological assessment among most at-risk populations in six high-prevalence states of India: design and implementation challenges. AIDS 22: S17-S34.

16. Karnataka Health Promotion Trust (2008) Integrated Biological & Behavioral Assessment: 2005

(round 1) and 2008 (round 2).

17. Karnataka Health Promotion Trust (2006) Sampling and design: Integrated behavioral and biological

assessment in Karnataka.

18. Karnataka Health Promotion Trust (2008) Female sex workers IBBA chapter report: Belgaum.

Bangalore, India.
