# Supplementary material for: HIV Epidemic Appraisals for Assisting in the Design of Effective Prevention Programmes: Shifting the Paradigm Back to Basics
Source: PLoS One. 2012 Mar 1;7(3):e32324. doi: 10.1371/journal.pone.0032324 (PMC3291609; doi:10.1371/journal.pone.0032324)
Supplement: Table S2 — Transmission dynamics epidemic classification. (DOC) [file pone.0032324.s003.doc]

**Table S2. Transmission Dynamics Epidemic Classification**

**Table S2. Source data for the Transmission Dynamics Epidemic Classification (TDEC) approach for six districts in India.**

|  | **District** | | | | | |
| --- | --- | --- | --- | --- | --- | --- |
|  | **Shimoga** | **Belgaum** | **Bagalkot** | **Ganjam** | **Sikar** | **Varanasi** |
| Overall HIV prevalence (year, source) | 1.0  (2008, PPTC)[1] | 1.43  (2007, GPS)[2] | 2.6  (2009,GPS)[3] | 3.25  (ANC,2008)[4,5,6] | 0.98  (GPS,2004)[7] | 0.25 (2008,PPTCT)[4,5,6] |
| Presence of a local HRG?[1,8] | Yes (FSW, MSM; NGO mapping)[1] | Yes (FSW, MSM; NGO mapping);  PAF (paid sex) among males 76.1% | Yes (FSW, MSM; NGO mapping); PAF (paid sex) among males 90.1% | Yes (FSW, MSM; NGO mapping)[9,10,11,12,13] | Yes (FSW, MSM; NGO mapping)[14,15,16] | Yes (FSW, MSM; NGO mapping)[8] |
| Connections with outside districts?[17,18] | Yes; but insufficient information to suggest paid sex at destination | Yes; client IBBA (higher HIV prevalence among men who travel out of district); FSW migration (IBBA)[19] | Yes; client IBBA (higher HIV prevalence among men who travel out of district); FSW migration (NGO program data)[1,19] | Yes; 11% male out-migration for short-term employment, anecdotal documentation of paid sex at destination district[13,20,21] | Yes; with evidence of paid sex at destination sites [7,14,15,18,22] | Yes; anecdotal evidence (NGO), ICTC clinic data [23](HIV prevalence among migrants 49.3% vs. 16.4% among non-migrant attendees) |
| Multiple partnerships in the general population? | No; key informants (NGO)[1] | Yes (10% of males; PBS) | Yes (5% males, 3⋅8% females; PBS in the rural region) | No; key informants (NGO)[9,10,11,12,21] | Yes; qualitative assessment of sexual behaviours of spouse who remains at home, similar HIV prevalence among males irrespective of migration status [14,15,18] | No; key informants (NGO)[23] |
| Classification | Concentrated - local | Concentrated (local & non-local) vs. Mixed | Concentrated (local & non-local) vs. Mixed | Concentrated – local & non-local | Concentrated – local & non-local vs. mixed | Concentrated – local & non-local |

NGO (non-governmental organization); PAF (population attributable fraction); HRG (high risk group); MSM (men who have sex with men); ANC (Antenatal clinic [sentinel surveillance]); PPTC(prevention of parent to child transmission clinic); GPS (general population survey[2,3]); PBS (polling booth survey[1]); IBBA (Integrated Biological and Behavioural Assessment[24,25,26]); ICTC (Integrated counselling and testing center). 2001 India Census Data [17] projected to 2006.

References

1. India Health Action Trust (2010) HIV/AIDS situation and response in Karnataka: Epidemiological

appraisal using data triangulation. Bangalore.

2. Rajaram S, Sangameshwar S, Jayachandran A, Bradley J, Alary M, et al. (2008) HIV and STIs in

Belgaum district, Karnataka, India. A general population survey. Bangalore. Available:

<http://www.khpt.org/charme.html>. Accessed 2 October 2011.

3. Rajaram S, Bradley J, Alary M, Ramesh B, Washington R, et al. (2010) HIV and STIs in Bagalkot

district, Karnataka, India. A general population survey. Bangalore. Available:

<http://www.khpt.org/charme.html>. Accessed 2 October 2011.

4. National AIDS control Organization (2009) Annual report 2008-2009. New Delhi: Ministry of Health

& Family Welfare, Government of India. Available:

http://www.nacoonline.org/Quick_Links/Publication/ME_and_Research_Surveillance/ . Accessed 2

October 2011.

5. National AIDS Control Organization (2010) UNAIDS Country Progress Report: India. Available:

<http://www.unaids.org/en/dataanalysis/monitoringcountryprogress/2010progressreportssubmittedbycountr>

ies/ . Accessed 2 October 2011.

6. National AIDS Control organization (2010) Press release: HIV declining in India. Delhi: Ministry of

health and family welfare, Government of India. Available:

http://www.nacoonline.org/Quick_Links/Publication/ME_and_Research_Surveillance/ . Accessed 2

October 2011.

7. Singh P (2010) Rajasthan State, Sikar, and Jalore district profile and HIV/AIDS data triangulation

report. Jaipur: India-Canada Collaborative HIV/AIDS Project.

8. India Health Action Trust (2010) HIV/AIDS situation and response in Uttar Pradesh: Epidemiological

appraisal using data triangulation. Bangalore.

9. GPSS (2007) Needs assessment survey of Digapahandi Block. Ganjam: Govinda Pradhan Smruti

Sansad.

10. GPSS (2008) Needs assessment survey of in-migrants in Ganjam district, India. Ganjam: Govinda

Pradhan Smruti Sansad.

11. GPSS and ARUNA (2010) Migration in Ganjam. Ganjam: Association for Rural Uplift and National

Allegiance and the Govinda Pradhan Smruti Sansad.

12. ARUNA (2006) Annual progress reports: Ganjam. Bhubaneshwar: Association for Rural Uplift and

National Allegiance.

13. Choudhury S (2010) Migration and vulnerability of HIV/AIDS in Ganjam, India. Bangalore:

Karnataka Health Promotion Trust.

14. Gelmon L, Ramesh B, Washington R, Singh K, Singh D, et al. (2005) The migrant household census

and biological baseline study from the Shekhawati region of Rajasthan - 2004. India Canada

Collaborative HIV/AIDS Project.

15. Gelmon L, Singh K, Singh P, Costigan A (2006) The sexual networking study from the out-migrant

community of the Shekawati region of Rajasthan: 2004-2005. Jaipur, India.

16. Raman Development Consultants (2009) Mapping of high risk groups and migratns in Rajasthan,

2009. Jaipur.

17. Government of India Ministry of Home Affairs (2001) Census India. Registrar General & Census

Commissioner, India.

18. Singh D, Costigan A, Moses S, O'Neil J, Blanchard J (2006) HIV programming for high out-

migrating rural populations. Rajasthan, India.

19. Buzdugan R, Halli S, Gaurav K, Satyanarayana, Blanchard JF (2009) Payana cohort of migrant and

mobile female sex workers in northern Karnataka: baseline results. Bangalore: Karnataka Health

Promotion Trust.

20. Governemnt of Orissa (2007) District statistical handbook of Ganjam. Bhubaneshwar: Directorate of

Economics and Statistics.

21. LEPRA (2004) Baseline survey of Aska and Hinjilikatu block. Ganjam: LEPRA: health in action.

22. Costigan A, Singh K, Singh P, Chatterjee P, Alix A (2005) HIV/AIDS and migration in Rajasthan.

Jaipur: India Canada Collaborative HIV/AIDS Project.

23. Singh A, Singh TB, Singh HK, Gulati AK (2010) Risk and significant factors associated with HIV

among elderly people attending Sir Sunderlal hospital, Banaras Hindu University, Varanasi. Varanasi:

Institute of Medical Sciences.

24. Karnataka Health Promotion Trust (2008) Female sex workers IBBA chapter report: Belgaum.

Bangalore, India.

25. Karnataka Health Promotion Trust (2008) Female sex wokers IBBA chapter report: Shimoga.

Bangalore, India.

26. Ramesh BM, Beattie TSH, Shajy I, Washington R, Jagannathan L, et al. (2010) Changes in risk

behaviours and prevalence of sexually transmitted infections following HIV preventive interventions

among female sex workers in five districts in Karnataka state, south India. Sex Transm

Infect 86 Suppl 1: 17-24.
